# Identification of mycobacteriophage toxic genes reveals new features of mycobacterial physiology and morphology

**DOI:** 10.1038/s41598-020-71588-5

**Published:** 2020-09-04

**Authors:** Ching-Chung Ko, Graham F. Hatfull

**Affiliations:** Department of Biological Sciences, University of Pittsburgh, Pittsburgh, PA 15260 USA

**Keywords:** Microbiology, Bacteriophages, Bacterial genetics

## Abstract

Double-stranded DNA tailed bacteriophages typically code for 50–200 genes, of which 15–35 are involved in virion structure and assembly, DNA packaging, lysis, and DNA metabolism. However, vast numbers of other phage genes are small, are not required for lytic growth, and are of unknown function. The 1,885 sequenced mycobacteriophages encompass over 200,000 genes in 7,300 distinct protein ‘phamilies’, 77% of which are of unknown function. Gene toxicity provides potential insights into function, and here we screened 193 unrelated genes encoded by 13 different mycobacteriophages for their ability to impair the growth of *Mycobacterium smegmatis*. We identified 45 (23%) mycobacteriophage genes that are toxic when expressed. The impacts on *M. smegmatis* growth range from mild to severe, but many cause irreversible loss of viability. Expression of most of the severely toxic genes confers altered cellular morphologies, including filamentation, polar bulging, curving, and, surprisingly, loss of viability of one daughter cell at division, suggesting specific impairments of mycobacterial growth. Co-immunoprecipitation and mass spectrometry show that toxicity is frequently associated with interaction with host proteins and alteration or inactivation of their function. Mycobacteriophages thus present a massive reservoir of genes for identifying mycobacterial essential functions, identifying potential drug targets and for exploring mycobacteriophage physiology.

## Introduction

The bacteriophage population is vast, dynamic, old, and highly diverse^1^. Phage genome sizes vary considerably from 5 to 500 kbp, but the average size of referenced phage genome sequences in publicly available databases is ~ 50 kbp^2^. The genome sequences of over 3,300 phages isolated on bacteria in the phylum Actinobacteria (i.e. ‘actinobacteriophages’) have been determined, with an average genome length of 61 kbp and an average of 100 predicted protein-coding genes per genome^3^. With an average gene length of about 600 bp, phage genes are substantially smaller than the average bacterial gene, reflecting an abundance of relatively small genes coding for non-structural proteins^3,4^. Phage genomes are characteristically mosaic as a consequence of horizontal genetic exchange^5,6^, although the rates of exchange vary for different types of phages^7^. Mosaicism is likely driven largely by microbial dynamics involving bacterial resistance to infection and phage co-evolution^8^.

The 3,300 sequenced actinobacteriophages include over 320,000 predicted protein coding genes, of which ~ 75% have no predicted function. Many of such genes are likely to be non-essential for viral lytic replication^9^, although most of them are expressed during lytic growth^10^. The general roles for these proteins may be in modulating the dynamic interactions with their host, counteracting restriction, CRISPR-Cas, and other viral defense systems^11–13^, or to actively promote defense against heterotypic viral attack^11,14–16^. Elucidating the roles and functions of these genes is of importance in understanding this biological dark matter^17^, and in the development and application of phages as therapeutic agents^18–20^.

A variety of phage genes have been shown to be toxic to their bacterial host when expressed outside of their native viral context. Examples include phage T4 *asiA*, which encodes an anti-σ^70^ factor that binds tightly to σ^70^ and inhibits *Escherichia coli* growth^21,22^, phage T7 gp2, which inactivates RNA Polymerase^23,24^, and the phage T7 Kil protein that interrupts FtsZ ring formation^25^. A variety of toxic genes have been reported using genomic screens, such as for *Rhodococcus* phage Yf1 and *Staphylococcal* phages^26,27^, and this has been proposed as a strategy for identifying novel drug targets^26^. Some mycobacteriophage-encoded genes have been shown to be toxic including mycobacteriophage L5 genes *77*, *78*, and *79*^28,29^, the product of one of which (gp77) interacts directly with the host protein Msmeg_3532^30^, and another (gp79) which promotes filamentation^28^.

In general, toxicity resulting from phage lytic gene expression is unlikely to be directly beneficial to the phage as they need metabolically active cells to support viral replication; moreover, cells will die due to lysis at the conclusion of lytic growth regardless. However, there are a variety of mechanisms by which phage-encoded proteins could interact with host proteins or complexes such as to be beneficial to the phage, and which are manifested as toxicity when the protein is experimentally overexpressed. One example is provided by mycobacteriophage Fruitloop gp52, which interacts directly with the host DivIVA (Wag31) protein, interferes with its function, and impedes superinfection by a DivIVA-dependent phage, Rosebush^31^. DivIVA is an essential protein, and although Fruitloop gp52 is toxic, it is the exclusion of Rosebush and not its toxicity per se that is beneficial to phage Fruitloop^31^. Phage-encoded gene toxicity thus provides a surrogate indicator for elucidating gene function and identifying interactions with the host metabolic machinery.

Here we screened 193 mycobacteriophage genes to identify those that inhibit host growth. We observe that ~ 25% negatively impact host cell growth when expression is induced, although the effects range from mild to severe. Together, these confer a variety of changes in growth and cellular morphology of *M. smegmatis*, including filamentation, and branching, as well as differential effects on survival of daughter cells at division. Identification and characterization of mycobacteriophage-encoded toxic genes provides an effective approach for understanding mycobacterial physiology and identifying potential new drug targets.

## Results

### Identification of mycobacteriophage-encoded toxic and inhibitory genes

Mycobacteriophages are genomically diverse and can be readily grouped into ‘clusters’ according to their overall sequence relationships^32–34^. Currently, there are 29 clusters (Clusters A, B, C, etc. to Cluster AC) and ten singleton phages (each with no close relative)^3^; all but one of these clusters (Cluster C, myoviridae) have siphoviral morphotypes. For the mycobacteriophage siphoviruses, the virion structure and assembly genes are syntenically organized and transcribed rightwards in the left parts of the genomes^4^. Genes in the right parts of the genomes have non-structural roles, are small relative to the structural genes, abundant, and most are of unknown function^4^.

To choose genes from the actinobacteriophage database to test for cytotoxicity (Table S1) we used the following criteria. First, we excluded virion structure and assembly genes, as well as the lysis and immunity genes. Second, we selected genes that are either of unknown function, or have non-specific functional assignments (e.g. DNA binding). Third, most of the genes are small (< 350 codons), and we specifically excluded larger non-structural genes whose functions are readily predicted (e.g. DNA Polymerases). Fourth, we generally chose genes from different protein ‘phamilies’ (phams) to avoid sequence redundancy. Fifth, the genes are either predicted or have been shown to be expressed during lytic growth. Sixth, we screened genes for the predicted likelihood of crystallization using PPCpred^35^ to facilitate downstream analysis. We screened genes from a diverse set of phages, but included larger numbers from phages LHTSCC, Fruitloop, and Wildcat (Table S1). Using these criteria, we selected 193 genes from 13 genomes from different clusters/subclusters for characterization of toxicity (Table S1).

**Figure 1:**
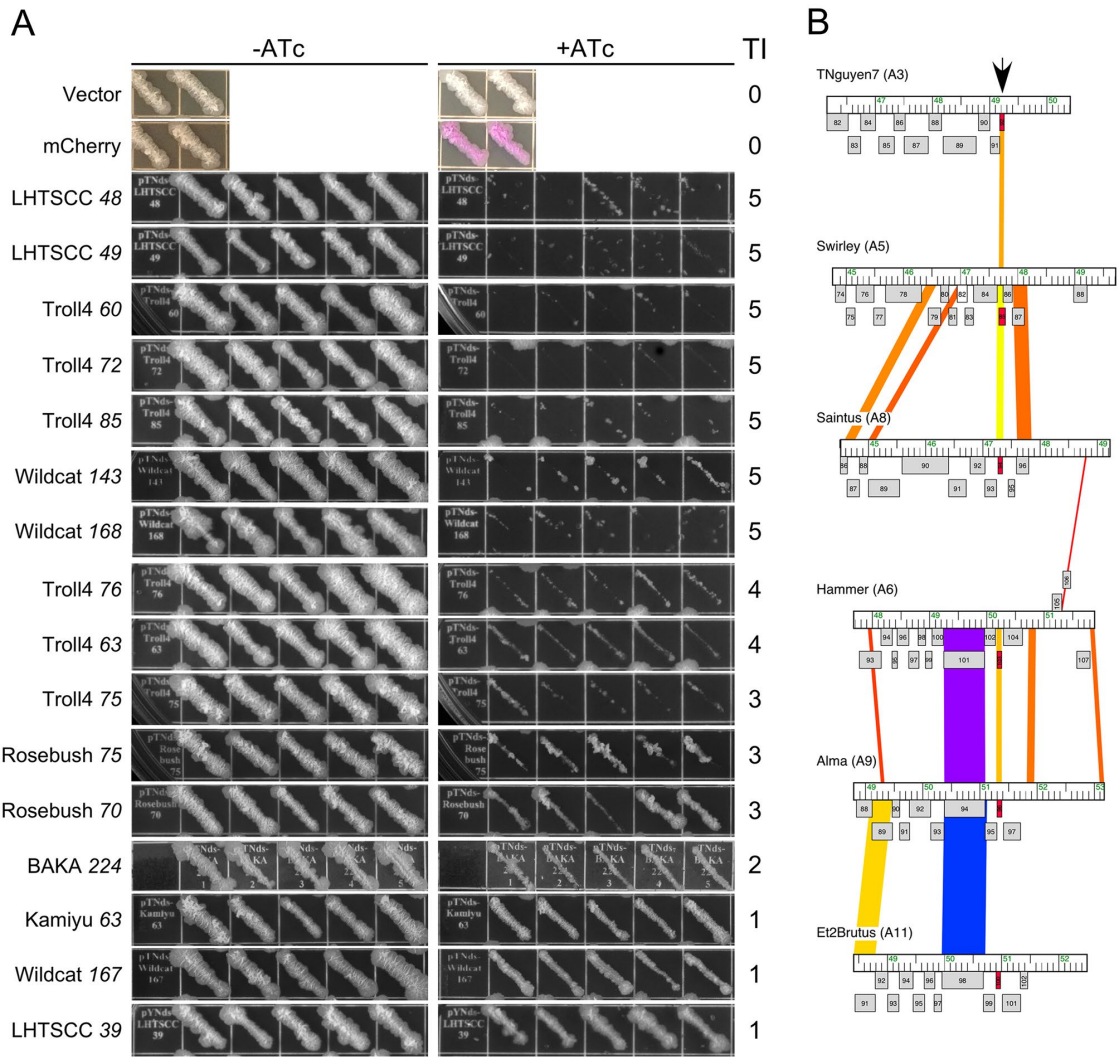
Identification of mycobacteriophage toxic genes. (**A**) Mycobacteriophage genes were cloned into an extrachromosomal vector under the control of a Tet-ON promoter and transformed into *M. smegmatis* mc^2^155. Five colonies of each were patched onto solid media with (+) or without (−) ATc. The toxicity of each gene was scored on a Toxicity Index (TI) from 0–5, with 0 and 5 corresponding to no toxicity and strong toxicity, respectively; shown is a subset of the tested genes. Two patched transformants with vector alone or with an mCherry-expressing plasmid are shown. The toxicity of all genes tested is listed in Table S1 and patches of all toxic genes are shown in Figs. S1 and S2. (**B**) Conservation of the 26 codon Hammer *103*. Segments of six Cluster A phage genomes are shown with the subcluster designation in parentheses following the phage name. Genes are shown as grey boxes above or below each genome corresponding to rightwards- and leftwards-transcription. Pairwise nucleotide sequence similarities are shown as spectrum-colored shading between genomes with violet corresponding to greatest similarity. Hammer *103* and its homologues are colored as red boxes, and indicated by the vertical arrow.

Each of the 193 genes were PCR amplified—including the open reading frame (ORF) and 30 bp upstream of the predicted translation initiation site to include the native ribosome binding site—and inserted into plasmid vector pTNds^31^, an extrachromosomal *E. coli*-mycobacterium shuttle vector with a Tet-ON promoter. Recombinant plasmids were recovered and verified in *E. coli*, transformed into *M. smegmatis* mc^2^155, and grown on solid media lacking the anhydrotetracycline (ATc) inducer of the Tet-ON promoter. For one gene—LHTSCC *91*—no *M. smegmatis* transformants were recovered, although the gene was successfully inserted into an integration-proficient vector (pTNdi^31^) from which transformants were recovered and used for subsequent analyses. For each plasmid, five individual colonies were picked and streaked onto plates with and without 100 ng/ml ATc (300 ng/ml ATc for LHTSCC *91*) and incubated at 37 °C (Fig. 1A). Transformants with vector only, or with a plasmid in which *mCherry* is fused to Tet-ON, were used as controls.

For 148 of the 193 (76.7%) genes tested, we saw similar growth in the presence and absence of ATc and no toxicity was observed; the other 45 genes (23.3%) showed varying levels of reduced growth in the presence of ATc (Fig. 1A, Table 1, Table S1, Figs. S1, S2). We scored the levels of toxicity in the presence of ATc using a Toxicity Index (TI; 0–5) with 5 being the most toxic and 0 indicating no toxicity (Fig. 1A, Table 1, Table S1, Figs. S1, S2). For one gene (Fruitloop *94*), toxicity was relatively mild, but bacterial growth on solid media containing ATc inducer has an altered morphology and appears smooth relative to the rough parent strain (Fig. S2). For some genes (e.g. Wildcat *145* and Wildcat *147*) poor growth was seen in the absence of inducer, likely indicating leaky expression of highly toxic genes in the absence of induction. For Wildcat *147* this was sufficiently severe that further propagation in liquid culture resulted in overgrowth by non-toxic mutants, and Wildcat *147* was not studied further. Overall, almost one quarter of the genes tested are deleterious to *M. smegmatis* growth when expression is induced with ATc.

**Table 1:** Mycobacteriophage toxic genes.

Phage (cluster), *gene*	aa	TI^1^	Bacteri- cidal^2^	Morphology^3^	Ms (top hit)^4^	Features^5^
LHTSCC (A4) *39*	52	1	NT	NT	No	Lipoprotein
LHTSCC (A4) *46*	56	5	Partial	Bulbous pole	Yes (Msmeg_6227)	NKF
LHTSCC (A4) *48*	100	5	Yes	Filament	No	DNA binding
LHTSCC (A4) *49*	57	5	Yes	Bulbous pole	Yes (Msmeg_1368)	Lipoprotein
LHTSCC (A4) *56*	25	2	NT	NT	No	NKF
LHTSCC (A4) *76*	46	2	NT	NT	No	NKF
LHTSCC (A4) *83*	156	5	Yes	Filament	Yes	SprT-like
LHTSCC (A4) *89*	68	4	NT	NT	No	NKF
LHTSCC (A4) *91*	39	5	No	No change	No	NKF
Hammer (A6) *103*	26	5	Yes	No change	No	NKF
Rosebush (B2) *44*	129	5	No	No change	No	DUF4447
Rosebush (B2) *70*	74	3	NT	NT	No	NKF
Rosebush (B2) *75*	81	3	NT	NT	No	NKF
Kamiyu (B3) *63*	39	1	NT	NT	No	NKF
Troll4 (D1) *60*	302	5	Yes	Filament	No	DUF669
Troll4 (D1) *63*	176	4	NT	NT	No	Methyltransferase
Troll4 (D1) *72*	117	5	Yes	Filament	No	NKF
Troll4 (D1) *75*	71	4	NT	NT	No	DNA binding
Troll4 (D1) *76*	146	4	NT	NT	No	DUF732
Troll4 (D1) *85*	179	5	Yes	Filament	No	NKF
Fruitloop (F1) *57*	121	3	NT	NT	No	DNA binding
Fruitloop (F1) *60*	192	2	NT	NT	No	Rad52-like
Fruitloop (F1) *78*	94	2	NT	NT	No	NKF
Fruitloop (F1) *83*	139	4	NT	NT	No	NKF
Fruitloop (F1) *94*	61	1	NT	NT	No	NKF
Konstantine (H1) *66*	89	5	Yes	Filament	No	NKF
Babsiella (I1) *50*	354	4	NT	NT	No	FtsK domain
Babsiella (I1) *68*	47	4	NT	NT	No	NKF
BAKA (J) *17*	186	5	Yes	No change	No	M34 peptidase-like
BAKA (J) *224*	50	2	NT	NT	No	NKF
Wildcat (V) *11*	215	5	Yes	Death at division	Yes	Methyltransferase
Wildcat (V) *13*	77	5	No	No change	Yes (Msmeg_1285)	LexA-like
Wildcat (V) *22*	124	4	NT	NT	No	NKF
Wildcat (V) *143*	59	5	Yes	No change	No	NKF
Wildcat (V) *144*	185	5	Yes	Branched fil	Yes	NKF
Wildcat (V) *145*	55	5	Yes	Branched fil	Yes (Msmeg_1516)	NKF
Wildcat (V) *147*	92	5	NT	NT	No	NKF
Wildcat (V) *155*	134	2	NT	NT	No	NKF
Wildcat (V) *159*	105	2	NT	NT	No	NKF
Wildcat (V) *162*	117	4	NT	NT	No	NKF
Wildcat (V) *163*	121	5	Yes	Filament	No	NKF
Wildcat (V) *165*	91	5	No	Curved	Yes	NKF
Wildcat (V) *166*	128	5	Yes	Filament	No	NKF
Wildcat (V) *167*	121	1	NT	NT	No	NKF
Wildcat (V) *168*	97	5	No	Bulbous pole	No	NKF

^1^TI = Toxicity Index from 1 to 5, with 5 being the most toxic and 1 being the least toxic.

^2^Genes are defined as bactericidal if survival is reduced by 100-fold or more after 12 h incubation post-induction. *NT* not tested.

^3^Observed by bright field microscopy during toxic gene expression. NT, Tested; Bulbous pole, cell pole enlarged; Filament, cells become filamentous; Branched fil., cells form branched filaments; Curved, curved cells.

^4^Mass spectrometry analysis of HA-tagged toxic proteins’ co-immunoprecipitation (co-IP) targets. Details can be found in Table S2. The most prominent hits are listed here.

^5^Features are determined through bioinformatic analyses. NKF = no known function. Hits to domains of unknown function (DUF) are shown.

Approximately two-thirds of the 45 toxic genes have no predicted function, and the others have a diverse array of potential functions, including DNA binding, proteases, methyltransferases, lipoproteins, and other functions (Table 1). The toxic/inhibitory gene products vary in length from 25 amino acids (LHTSCC *56*) to 354 amino acids (Babsiella *50*). At only 26 and 25 amino acids respectively, the assignments of Hammer *103* and LHTSCC *56* (both phages are in Cluster A) warrant further examination (Fig. 1B). We note that there are 95 genes related to Hammer *103* (grouped in the same phamily) in the Cluster A phage genomes, distributed across four subclusters, located downstream of the P_left_ early lytic promoter, and expressed early in lytic growth^36,37^ (Fig. 1B). Homologues of Hammer *103* have been assigned to these genomes even though they are quite varied at the nucleotide sequence level (Fig. 1B). This comparative genomic analysis and the toxicity of these ultra-small genes supports their assignment in the genome annotations.

### Impact of toxic gene expression on *M. smegmatis* growth

**Figure 2:**
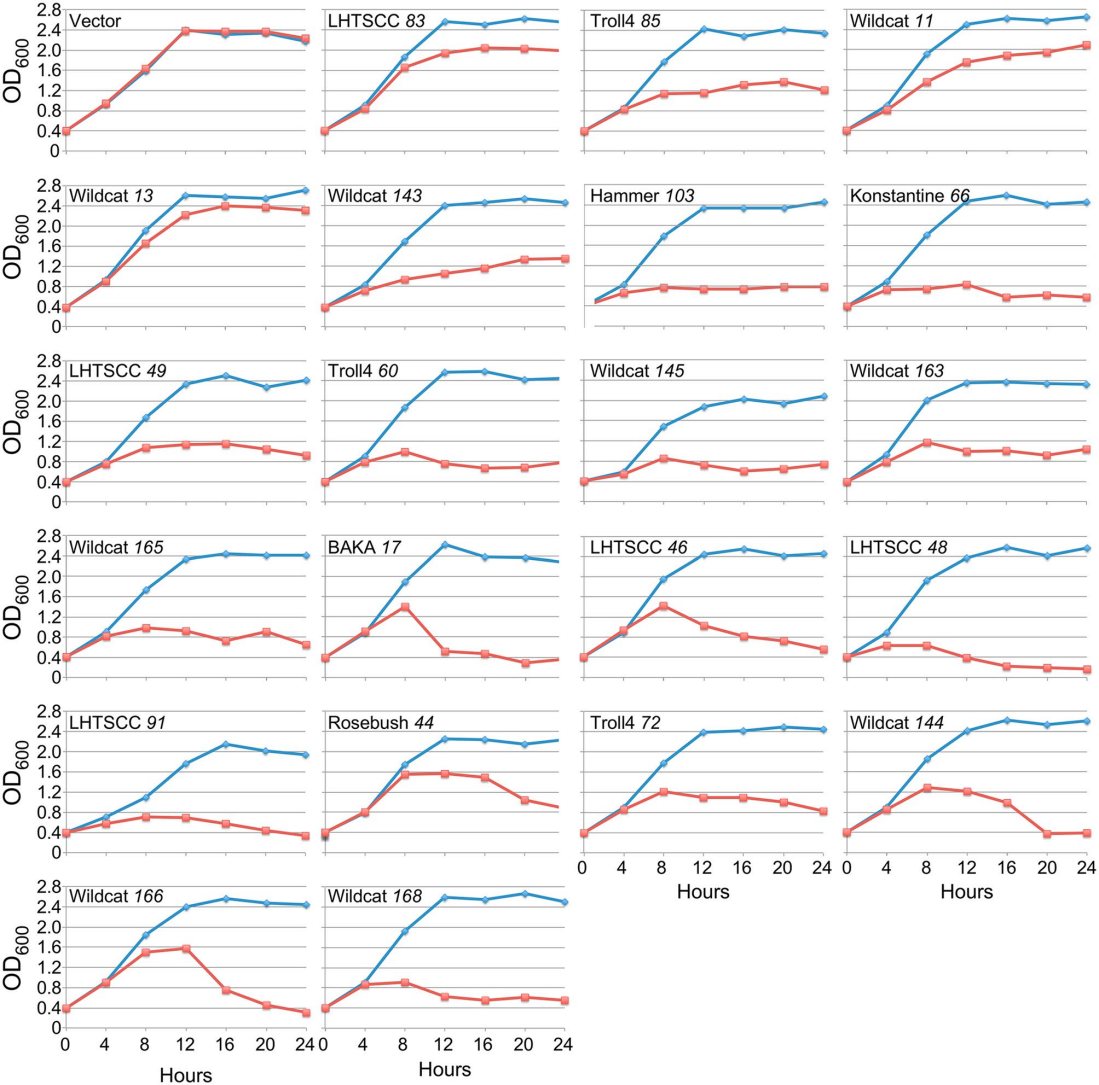
Growth of *M. smegmatis* following induction of toxic gene expression. *M. smegmatis* transformants carrying mycobacteriophage toxic genes were grown in liquid culture to OD_600_ of ~ 0.4, induced by addition of 100 ng/mL ATc at time 0, and OD_600_ measured at 4-h intervals. Blue and red lines indicate OD_600_ in the absence and presence of inducer, respectively. All genes are cloned into an extrachromosomally-replicating plasmid vector with the exception of LHTSCC *91* was inserted into an integration-proficient plasmid vector.

Twenty-one of the most-toxic genes (toxicity index = 5) were characterized for their impact on *M. smegmatis* growth when induced in liquid culture (Fig. 2). Protein expression was induced in each strain in early log-phase growth (OD_600_ at 0.4) and cell densities were measured every 4 h. For the 21 genes, a wide range of responses was observed (Fig. 2). Five genes (LHTSCC *83*, Troll4 *85*, Wildcat 11, Wildcat *13*, Wildcat *143*) show relatively mild defects with continued growth to varying extents at reduced rates over the course of the experiment. Seven genes show little or no increase in optical density after the 4-h (Hammer *103*, Konstantine *66*) or 8-h (LHTSCC *49*, Troll4 *60*, Wildcat *145*, Wildcat *163*, Wildcat *165*) timepoints, with similar OD values for the remaining time (Fig. 2). Nine of the genes (BAKA *17*, LHTSCC *46*, LHTSCC *48*, LHTSCC *91*, Rosebush *44*, Troll4 *72*, Wildcat *144*, Wildcat *166*, Wildcat *168*) show at least a 30% decrease in OD values after the 4–8 post-induction period, indicating either lysis or severe clumping (Fig. 2). For two genes (BAKA *17* and Wildcat *166*), the rapid reduction in OD within a four-hour window is particularly notable.

### Loss of viability with toxic gene expression

**Figure 3:**
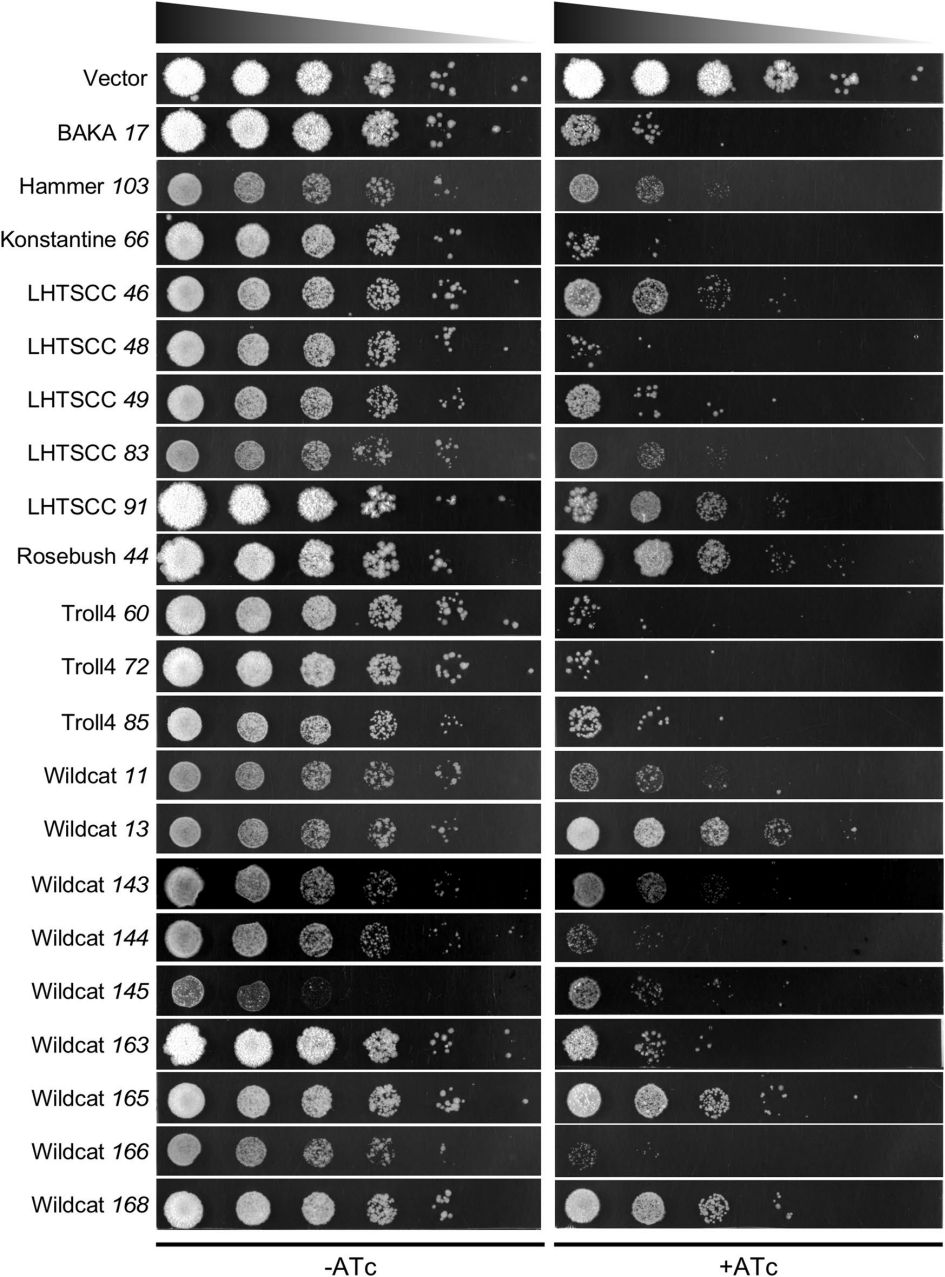
Survival and recovery after toxic gene induction. Twelve hours after addition of ATc inducer to liquid cultures, uninduced (−ATc) and induced (+ ATc) samples (as shown in Fig. 2) were washed and tenfold serial dilutions plated for growth on solid media without ATc.

Cellular toxicity could arise either from a temporary cessation of growth, or from irreversible loss of viability. We determined whether expression of the most toxic genes (Toxic Index = 5) irreversibly interferes with survival by growing liquid cultures for 12 h in either the presence or absence of inducer, and then plating for growth on solid media (Fig. 3). For five genes (LHTSCC *91*, Rosebush *44*, Wildcat *13*, Wildcat *165*, Wildcat *168*), all of which—with the exception of Wildcat *13*—strongly inhibit growth in liquid culture, there was little or no reduction in viability suggesting that toxicity largely is bacteriostatic, (Fig. 2). The other genes confer varying degrees of irreversible loss of viability, but in the most extreme examples (BAKA *17*, Konstantine *66*, LHTSCC *48*, Troll4 *60*, Troll4 *72*, Troll4 *85*, Wildcat *144*, and Wildcat *166*) viability is reduced by at least three orders of magnitude (Fig. 3). These include four genes (BAKA *17*, LHTSCC *48*, Wildcat *144*, and Wildcat *166*) that showed a marked reduction in OD_600_ in the liquid culture—with the OD_600_ reading at 24 h at or below the starting OD_600_—and four genes (Konstantine *66*, Troll4 *60*, Troll4 *72*, and Troll4 *85*) that have milder impacts on growth in liquid culture (Fig. 2).

### Impact of toxic gene expression on *M. smegmatis* morphology

**Figure 4:**
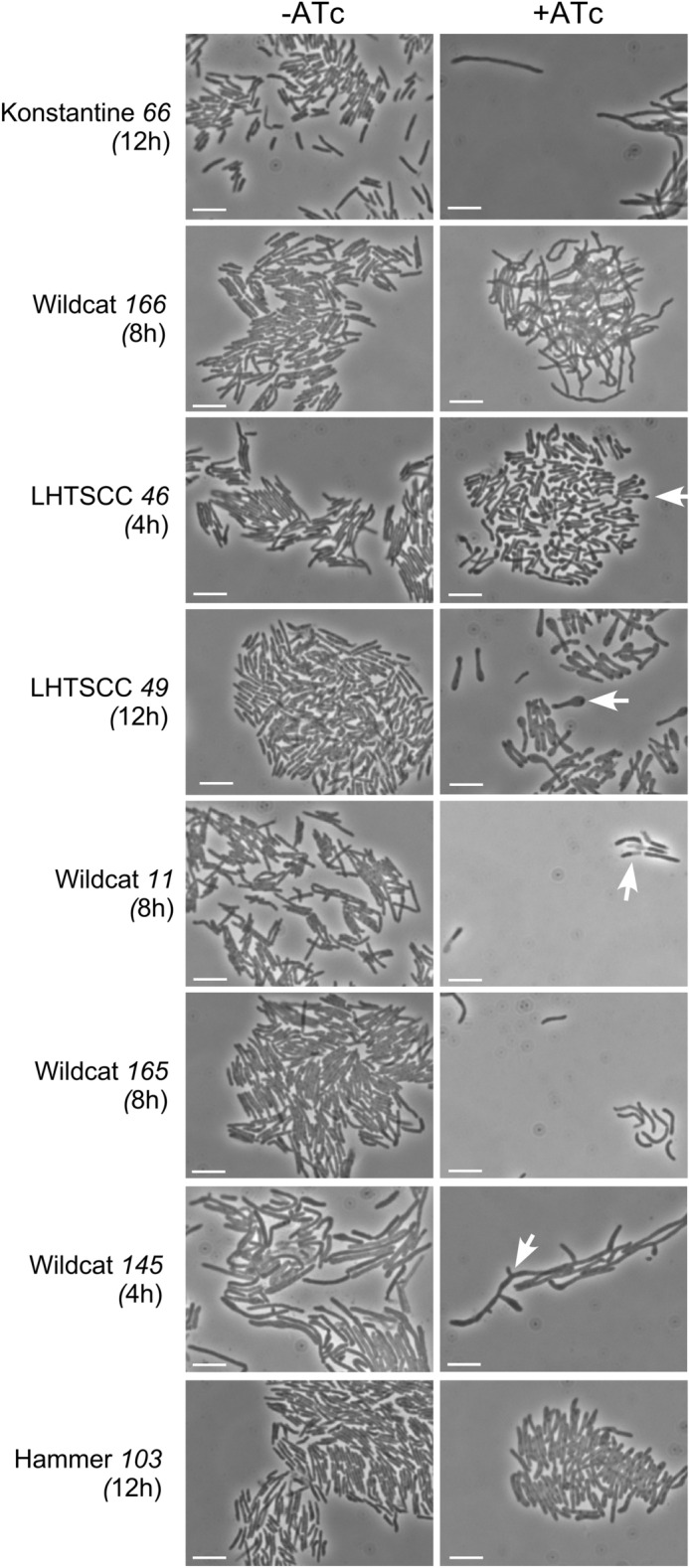
Changes in cellular morphologies following induction of toxic gene expression. *M. smegmatis* transformants carrying mycobacteriophage toxic genes were examined by bright field microscopy 4–12 h after induction (as indicated). The scale bar corresponds to 5 µm. Arrows indicate notable features including bulbous poles (LHTSCC *46*, LHTSCC *49*), branches (Wildcat *145*), and differential appearance of daughter cells (Wildcat *11*).

We examined changes in cellular morphologies in strains expressing the 21 most toxic genes (Toxic Index = 5) at, 0, 4, 8, and 12 h after addition of ATc inducer (Fig. 4, Table 1, Figs. S3–S6). For six genes (BAKA *17*, Hammer *103,* LHTSCC *91*, Rosebush *44*, Wildcat *13*, and Wildcat *143*) we observed no evident morphological changes, but the other 15 strains showed a variety of responses. A relatively common phenotype is filamentation, presumably from inhibition of cell division, and ten of the toxic genes (Konstantine *66*, LHTSCC *48*, LHTSCC *83*, Troll4 *60*, Troll4 *72*, Troll4 *85,* Wildcat *144*, Wildcat *145*, Wildcat *163*, Wildcat *166*) show this phenotype (Fig. 4, Figs. S3–S6). Expression of Wildcat *145* forms filaments containing branches, as does Wildcat *144* to a lesser extent (Fig. 4; Figs. S3, S4). Wildcat *166* expression appears to cause curved or crooked filaments (Fig. 4; Fig. S3). One gene (Wildcat *165*) does not obviously promote filamentation, but forms curved cells, perhaps by inhibiting cell wall growth unequally across the cell (Fig. 4; Fig. S4). Expression of three genes (LHTSCC *46*, LHTSCC *49*, Wildcat *168*) results in formation of bulbous ends at one pole of the cell (Fig. 4; Fig. S4), a phenotype reminiscent of expression of Fruitloop gp52, which is known to interact directly with *M. smegmatis* Wag31 (DivIVA)^31^.

**Figure 5:**
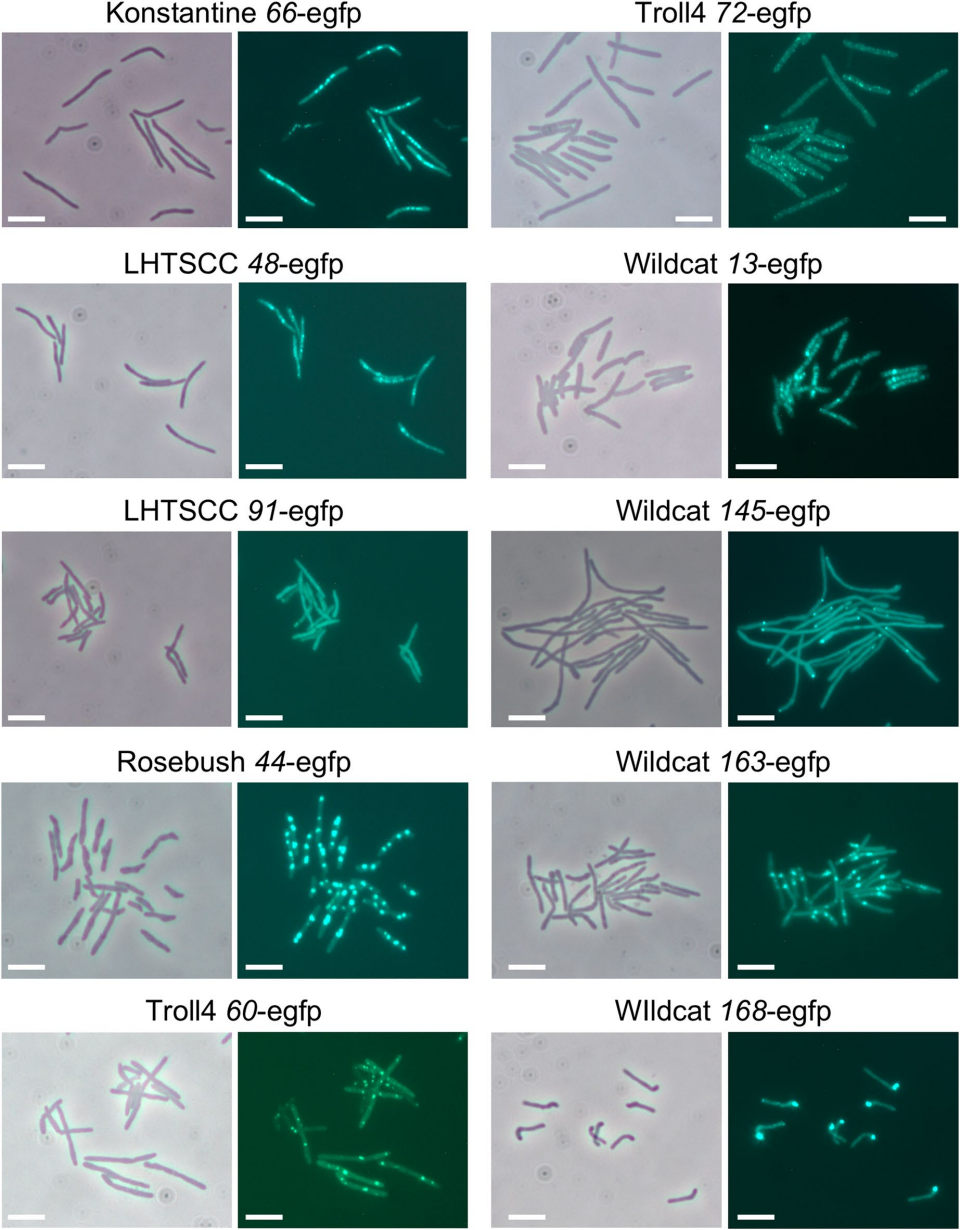
Localization of toxic proteins. Toxic genes were fused at their 3′ ends to egfp and tested for maintenance of the toxic phenotype (see Fig. S7). Those retaining toxicity were induced for 4 h and examined by fluorescent microscopy. The scale bars correspond to 5 µm.

To examine localization of the toxic proteins we constructed C-terminal eGFP fusions of each of the toxic proteins. First, we tested if the fusion maintains toxicity (Fig. S7); twelve were severely abrogated in their toxicity and were not analyzed further (i.e. the TI changed from 5 of the native protein to 0 or 1 for the fusion). The remaining ten fusions were examined by fluorescent microscopy 4 h after addition of ATc inducer (Fig. 5), and several notable patterns were observed. First, Wildcat gp168-eGFP appears to localize to the bulbous end of the cell (Fig. 5), and is reminiscent of the localization of Fruitloop gp52^31^. Second, six of the genes that promote filamentation (Konstantine *66*, LHTSCC *48*, Troll *60*, Troll4 *72*, Wildcat *145*, and Wildcat *163*) form fluorescent puncta, which with Wildcat gp145-eGFP are localized predominantly at one pole of the filament (Fig. 5). The other five strains have several distinct puncta arranged throughout the filament. Rosebush gp44 does not promote filamentation per se but forms 2–4 puncta in each cell (Fig. 5).

**Figure 6:**
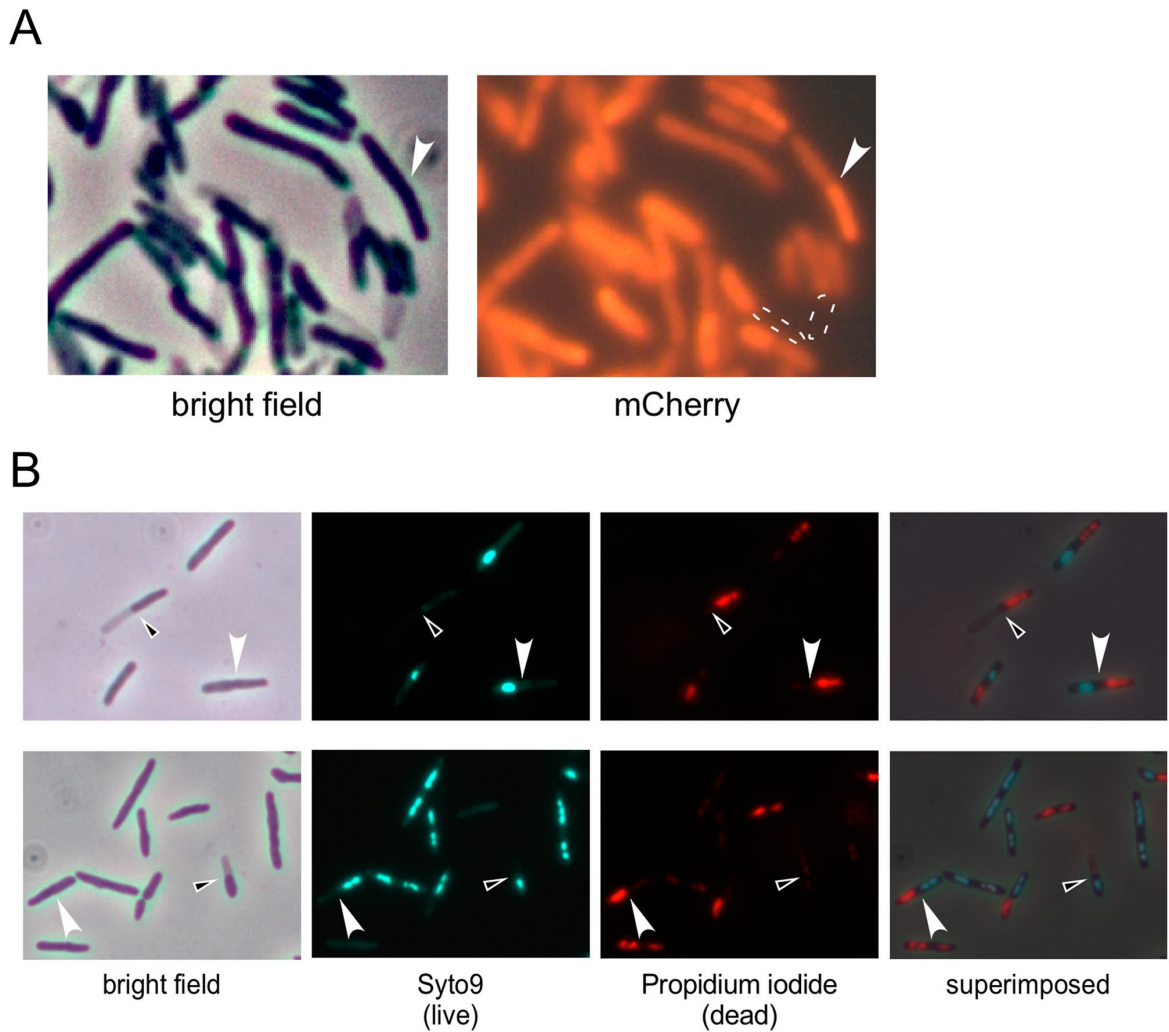
Asymmetric toxicity of Wildcat gp11 at cell division. (**A**) *M. smegmatis* carrying both an ATc-inducible Wildcat 11 plasmid and a constitutively expressed *mCherry* plasmid was induced for 5.5 h and examined microscopically. Several cell pairs were observed in which one cell appears normal, and other appears light. mCherry fluorescence is not observed in the light transparent-looking cells (dotted white outline). White arrows indicate an example where the two cells of a pair have different mCherry levels but are similar by light microscopy. (**B**) An *M. smegmatis* transformant expressing Wildcat *11* was induced for 6 h and stained with a Syto9/Propidium iodide mixture for live/dead staining. Several examples where each of two cells of a pair stain as either live or dead but look similar (white arrow) or different (black and white arrow) in the bright field are indicated.

One of the most intriguing morphological changes occurs by expression of Wildcat gp11 (Fig. 4). In bright field images, we observed many pairs of cells (2–20%; average of 10% in five images) in which one appears to be normal but the other has a ghost-like appearance (Fig. 4). In cells expressing mCherry (not as a fusion protein), only the more normal-appearing cell of each pair has normal fluorescence and the partner cell has little or no fluorescence (Fig. 6A). The different fates of *M. smegmatis* daughter cells at division has been described previously^38^, and we propose that as the cells divide, Wildcat gp11 causes death in just one of the two types of daughter cells. Consistent with this, we find that live/dead staining shows instances in which one cell of a pair stains positively with Syto9 and is alive, whereas the partner cell stains with propidium iodide and is dead (Fig. 6B); in some cell-pairs, one partner cell stains with propidium iodide, and the other does not stain at all, perhaps indicating complete loss of DNA (Fig. 6B). We note that for some cell pairs, the daughters stain differently but appear similar by bright field microscopy (Fig. 6).

### The Wildcat gene *143* to *171* region is rich in toxic genes

**Figure 7:**
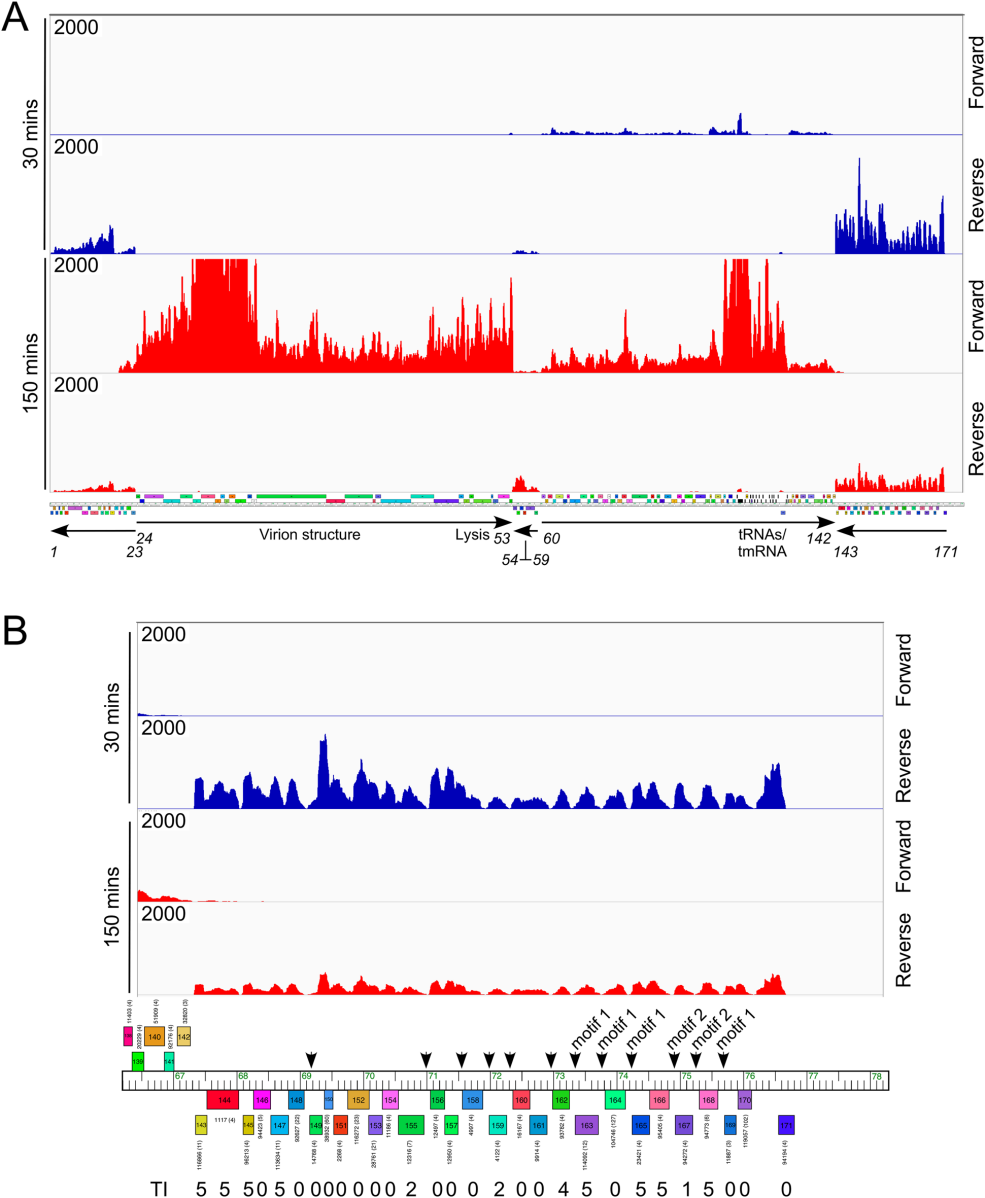
RNA sequencing of Mycobacteriophage Wildcat lytic growth. (**A**) *M. smegmatis* was infected with mycobacteriophage Wildcat at a multiplicity of infection of 3 and total RNA was isolated 30 min and 150 min post infection. Strand-specific sequence reads were mapped to the Wildcat genome with rightwards and leftwards transcripts indicated as ‘Forward’ and ‘Reverse’, respectively. Thirty and 150 min read counts are shown in blue and red, respectively. The Wildcat genome map is shown with arrows indicating the direction of transcription of groups of genes, and the gene numbers at the ends of these groups are indicated. (**B**) Genome organization and transcription at the extreme right end of the Wildcat genome. Genes *143*–*171* are leftwards-transcribed early in lytic growth, all were screened for toxicity, and the Toxicity Index (TI) is shown below. Many of the genes are separated by 80–100 bp non-coding intergenic DNA (arrows), some of which contain conserved motifs (motif 1 and motif 2). The RNAseq data is visualized using Integrative Genomics Viewer (version 2.3.92)^51^.

The Wildcat genome has a set of 29 leftwards-transcribed genes at the extreme right end of its genome (Fig. 7). Because these have the characteristics of being small and all are of unknown function, we tested all of these for toxicity (Table S1). Twelve of these (41%) showed some degree of toxicity, two-thirds with a Toxicity Index of 5 (Table S1). Because toxic genes may be involved in influencing phage-host dynamics including exclusion—as exemplified by Fruitloop *52*^31^—we predict that these are expressed early in lytic growth. To examine this in Wildcat, we performed RNAseq analysis at early and late lytic growth (30- and 150-min post infection, respectively; Fig. 7A). We observed distinct gene expression patterns at the early and late time points, with four blocks of genes expressed at 30 min: genes *60*–*142* (rightwards transcribed), *1*–*23*, *54*–*59*, and *143*–*171* (all leftwards-transcribed). Genes *143*–*171* are the most highly expressed early genes. By 150 min, the *143*–*171* and *1*–*23* regions have lower RNA levels, and there is higher expression of the *60*–*142* region. The virion structural genes (*24*–*45*) and the lysis cassette (*49*–*52*) are only expressed at the 150 min time point, and the capsid (*30*) and major tail (*35*) subunits are among the most highly expressed genes (Fig. 7A). The most highly expressed lytic gene is the phage tmRNA gene, which has a 15-fold higher RNA signal than the virion structural genes.

Because of the prevalence of toxic genes in the *143–171* region, we bioinformatically examined this region further. The genes lack the typical organization of closely-packed ORFs in an operon with closely-spaced translation start and stop codons (Fig. 7B)^39^; for example, in the *155* to *170* segment, 11 of the 15 intergenic distances are 80–100 bp long, and likely contain regulatory sequences. We have not been able to identify any single conserved sequence motif common to these regions, although there is a 60 bp region (Motif 1) shared (75% identity) in the four gene intervals *162*–*163*, *168*–*169*, *163*–*164*, and *164*–*165* (Fig. S8), and a second 45 bp motif (Motif 2; 82% identity) in the two intervals *166*–*167* and *167*–*168* (Fig. S8). We also note that the *143*–*171* region is evidently mosaic in structure, with 12 of the 29 genes having homologues in a variety of phages in other clusters, including *Gordonia*, *Arthrobacter*, *Microbacterium*, and *Streptomyces* phages; typically only a single gene is shared with different flanking genes to the left and to the right. Finally, the RNAseq of this region shows a shark-tooth appearance, with low points between genes that correspond closely with the regions where there are small (~ 100 bp) intergenic gaps. Although we’ve not been able to identify putative promoter or terminator sequences, many of these genes may be independently expressed.

The Wildcat *143*–*171* gene series is separated from the *1*- *23* genes—which are also leftwards transcribed (Fig. 7A)—by *cos*. During lytic growth when the *cos*-ends are ligated, transcription does not appear to traverse *cos*, and the region between gene *171* and *cos* is not transcribed (Fig. 7B). However, genes *1*–*23* are also expressed early in lytic growth (Fig. 7A), although the genes are not separated by intergenic non-coding regions (with the exceptions of *3–4* and *18–19* which are separated by ~ 50 bp gaps). Fourteen of the 23 genes were tested for toxicity, but only Wildcat *11*, *13* and *22* are toxic. Thus, although the *1–23* and *143–171* are expressed early in lytic growth, they appear to be organizationally and functionally distinct.

### Identification of interacting proteins by co-immunoprecipitation

**Figure 8:**
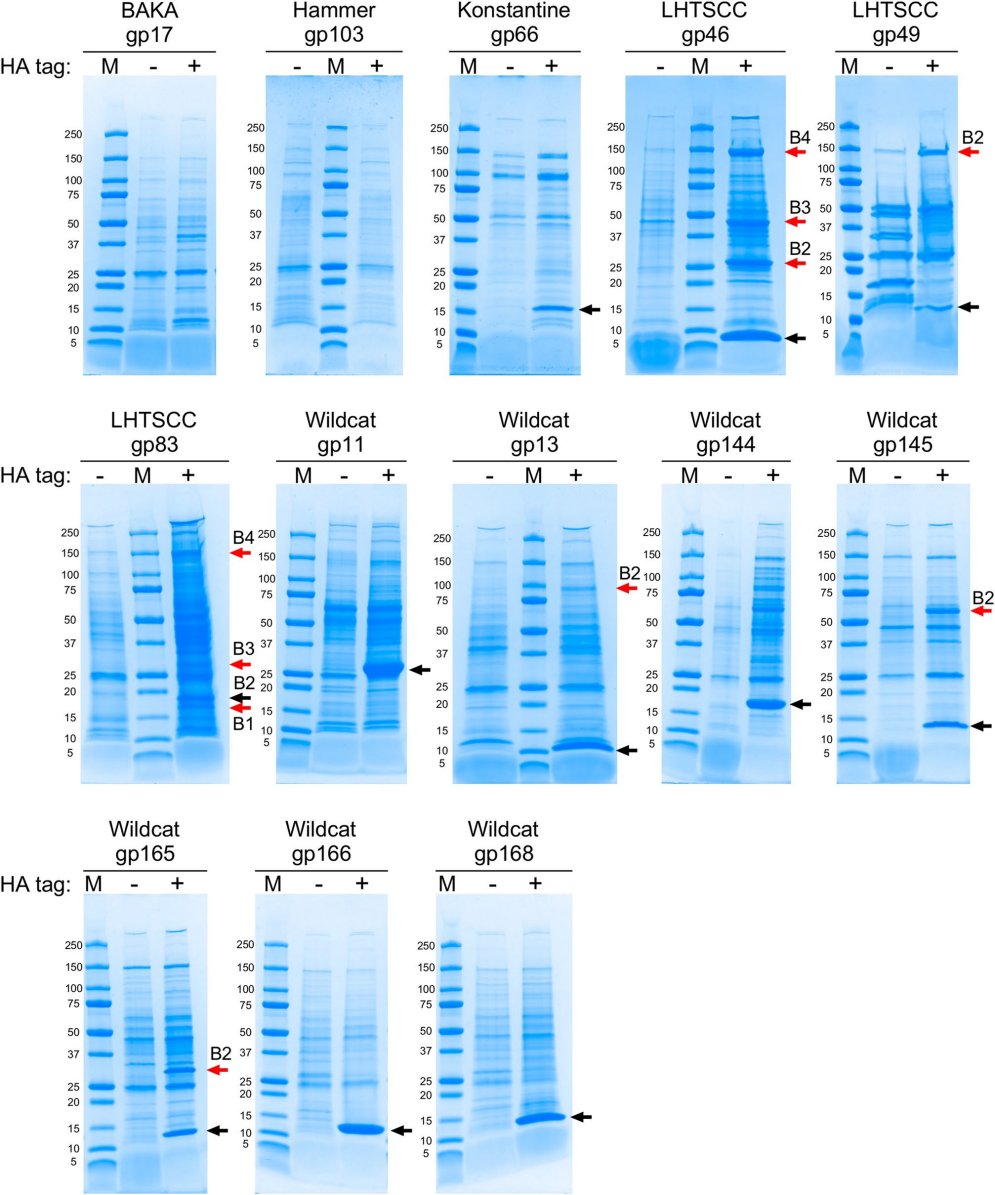
Co-Immunoprecipitation of proteins interacting with toxic proteins. HA-tags were added to thirteen of the toxic proteins, expressed in *M. smegmatis*, and immunoprecipitated using anti-HA. Co-immunoprecipitating (co-IP) proteins were recovered and separated by 4–20% gradient polyacrylamide gel electrophoresis (+). Controls for each protein used the same toxic protein but lacking the HA C-terminal tag (−). Protein size markers (M) are shown in kDa. The black arrows indicate a protein band corresponding to the predicted size of the toxic protein, and the red arrows with the corresponding labels indicate protein bands characterized by mass spectrometry (Table S2). For Wildcat gp165, band B2 was identified predominantly as Wildcat gp165-HA migrating at a position corresponding to a Wildcat gp165 dimer. Uncropped images are shown in Fig. S9.

The patterns of protein localizations, cellular morphologies, bacterial growth, and irreversible killing suggests that the toxicity of many of the toxic proteins results from specific interactions with host components, rather than non-specific effects. To explore this, we constructed HA-tagged derivatives of 13 toxic proteins, expressed them in *M. smegmatis*, and analyzed the immunoprecipitated proteins by SDS-PAGE (Fig. 8). Strong elution conditions were used to try and capture as many interacting proteins as possible^31^, although this yields a high background of protein recovery in control experiments in which the phage proteins lack the HA tag (Fig. 8). Nonetheless, several of the toxic proteins appear to co-precipitate with relatively prominent protein bands that are absent in the controls, notably with LHTSCC gp46, LHTSCC gp49, LHTSCC gp83, Wildcat gp13, Wildcat gp144, Wildcat gp145, and Wildcat gp165 (Fig. 8).

Co-immunoprecipitating proteins were identified by mass spectrometry, either by excising individual protein bands or by analysis of the whole protein eluate (Table S2). For several of the toxic proteins, strong predictions for interacting proteins can be made. First, for LHTSCC gp46, three prominent co-precipitating bands were observed with apparent sizes of ~ 27 kDa, ~ 48 kDa, and ~ 150 kDa (Fig. 8). Analysis of the 27 kDa showed that it is predominantly a PadR-family transcriptional regulator (Msmeg_6227), and these spectra were 100-fold more abundant in the HA-tagged sample than the non-tagged sample (Table S2). The most abundant peptides in the 48 kDa band correspond to EF-Tu, but are only represented 2.5-fold over the non-tagged control; it is a highly abundant protein in *M. smegmatis* and it may not reflect a specific interaction (Table S2). The 150 kDa band has β’ subunit of RNA Polymerase and is especially enriched relative to the non-tagged control sample. The β subunit of RNA polymerase is also detected in the whole protein eluate of the HA-tagged sample with ~ five-fold more peptide spectra than the non-tagged sample. Together, these data suggest that LHTSCC gp46 interacts with the gene expression machinery, and possibly with Msmeg_6227 itself. Interestingly, analysis of the entire eluate precipitated with Wildcat gp11 also showed Msmeg_6227 peptides recovered from the HA tagged protein but not the non-tagged control (Table S2). Msmeg_6227 is at similar molecular weight to Wildcat gp11 and likely co-migrates in SDS-PAGE (Fig. 8). We note that the closest relative of Msmeg_6227 in *M. tuberculosis* is Rv3488^40^, which shares weak similarity (35% amino acid identity) in a central 85-residue segment of the protein. Rv3488 has been implemented in countering toxic metal stress^40^.

For Wildcat gp145, a co-immunoprecipitating band migrating at approximately 60 kDa was excised from the HA-tagged sample (and from an equivalent position in the non-tagged sample) and analyzed by mass spectrometry. Peptides were identified that correspond to many *M. smegmatis* proteins—most with predicted molecular weights between 55 and 65 kDa—and most with similar numbers of peptides in the two samples. However, thioredoxin disulfide reductase (Msmeg_1516) has by far the greatest number of spectra and likely reflects the prominent band observed by SDS-PAGE (Fig. 8; Table S2). Msmeg_1516 is a homologue of Rv3913 (36% amino acid identity) that is implicated in stress tolerance^41^.

A large number of proteins co-precipitated with LHTSCC gp83-HA, and several excised bands predominantly contain spectra from ribosomal proteins (Table S2). LHTSCC gp83 may thus interact with ribosomes, but whether this is specific and related to its toxicity is unclear. Likewise, a large molecular weight band co-precipitating with LHTSCC gp49-HA was identified as the β’ subunit of RNA Polymerase, but the specificity of the interaction is unclear. Finally, a prominent band co-precipitating with Wildcat gp13 was identified as Msmeg_1285 (Fig. 8; Table S2). It may reflect a specific interaction, and Msmeg_1285 is a putative scaffolding protein that interacts with PonA1 a putative D,D-transpeptidase^42^. Msmeg_1285 is a homologue (59% amino acid identity) of *M. tuberculosis* Rv0613c, which is non-essential for in vitro growth of *M. tuberculosis* H37Rv and is reported to be membrane-associated^43^.

## Discussion

Like most bacteriophages, mycobacteriophage genomes have large numbers of genes of unknown function. Determining their roles in phage life cycles is complicated by the observation that many are not required for lytic growth^9^. They are also often not conserved among groups of closely-related genomes, although they frequently contribute to mosaic relationships and have relatives in unrelated genomes^2^. Screening genes for toxicity when expressed in the host is a relatively simple way of observing particular phenotypes and identifying potential interacting host protein partners^26,31^. Here, screening of 193 diverse mycobacteriophage genes from 13 different genomes showed that about 23% are toxic when expressed in *M. smegmatis*. We note that the expression system used is tightly, but not completely repressed in the absence of inducer, and genes with severe toxicity are difficult to recover and propagate (Figs. S1, S2). It is also plausible that some genes may be expressed at sufficiently high levels when fully induced that they confer non-specific toxicity that is not informative. Nonetheless, a range of toxicity was observed, and the genes can generally be classified as having severe or mild effects on bacterial growth.

Non-specific toxicity is a general concern when screening phage genes for inhibitory effects. However, many of the genes tested here result in irreversible growth inhibition and changes in cellular morphology that suggest interference with cell division, or growth at the cell poles, all of which are likely to reflect specific effects. The most striking of the morphological effects is the apparent impairment by Wildcat gp11 of one cell of a daughter pair after division, reflecting a highly specific consequence. The co-immunoprecipitation studies agree with these interpretations and support that toxicity often results from protein interactions. Confirmation of these interactions will require reverse co-IP with tagged target proteins, and to-date this analysis has confirmed only the interactions between Fruitloop gp52 and Wag31^31^ and Wildcat gp13 with Msmeg_1285 (our unpublished observations).

One of the most intriguing phenotypes observed resulted from expression of Wildcat gp11. Wildcat gp11 is a 25 kDa protein containing a methyltransferase motif (Pfam: Methyltranf_24 PF13578), is strongly toxic on solid media, and confers largely irreversible mortality when induced in liquid culture, even though there is only a modest impairment in OD_600_ rise. Microscopy shows that pairs of cells—presumably reflecting daughter cells immediately after division—contain only one cell that stains as being alive. It is known that at division one daughter cell inherits a growing pole whereas the other has to establish a growing pole^38^ and it seems plausible that Wildcat gp11 can distinguish between these two types of cells. Co-immunoprecipitation suggests that Wildcat gp11 interacts with the PadR-like transcriptional regulator, Msmeg_6227 (Fig. 8), which has been shown to be highly abundant in dormant *M. smegmatis* cells^44^. It is not clear whether Msmeg_6227 itself localizes differentially between daughter cells at division, or if it regulates expression of genes involved in these processes. Msmeg_6227 is not essential for viability of *M. smegmatis*^45^, so it is plausible that Wildcat gp11 modulates its regulator properties, rather than simply inactivating it. Whether the methyltransferase activity of Wildcat gp11 is required for the toxic and morphological effects is not known.

Interestingly, LHTSCC gp46—a 56-residue protein with no conserved domains and no known function—also interacts with Msmeg_6227 (Fig. 8). LHTSCC gp46 expression confers a different morphological change than Wildcat gp11, resulting in a prominent bulge at one pole of the cell, which we presume to be the growing pole (Fig. 4). LHTSCC gp46 might confer a different type of Msmeg_6227 dysregulation than Wildcat gp11, resulting in the different morphologies. How phage infection by either LHTSCC or Wildcat might benefit from dysregulation of Msmeg_6227 is unclear, but it is possible that it results in exclusion of phages that hypothetically might require Msmeg_6227 for efficient infection, mirroring the activity of Fruitloop gp52, which induced a similar morphology and also localizes to the pole^31^.

Curiously, Wildcat 1*65* expression promotes cell curvature. Wildcat gp165 is a 91-residue protein of unknown function that is very toxic, but mostly bacteriostatic when induced in liquid culture. We note that similarly curved cells were observed when the host DivIVA (Wag31) gene was C-terminally tagged with eGFP^46^. This is a phenotype distinct from the bulging poles observed when DivIVA is depleted^47^ or when Fruitloop gp52 is expressed^31^. We have not been able to show a direct interaction between Wildcat gp165 and Wag31, but this may represent a second instance in which two unrelated phage proteins (Fruitloop gp52 and Wildcat gp165) target the same host protein (Wag31) but in different ways, with different morphological outcomes.

We note that phages such as Wildcat may have large groups of genes such as the *143*–*171* group that are expressed early and perhaps influence host-phage dynamics. There are many genes in this group that are toxic when expressed in *M. smegmatis*, and it is plausible that the non-toxic genes may also modulate host processes, but in ways that do not impair cell growth or survival when overexpressed. Toxic genes identified in screens such as those described here may thus be useful for characterizing the functions of genes that are closely linked and similarly expressed.

Finally, this report suggests that ~ 25% of non-virion mycobacteriophage genes have a toxic phenotype, and that a deeper investigation could provide a very large number of toxic genes, many with informative interactions with host proteins. The ~ 1,880 sequenced mycobacteriophage genomes contain ~ 200,000 genes constituting over 15,000 phamilies, of which 3,000 may have toxic phenotypes. Further characterization of these genes and their interacting protein partners will provide new insights into mycobacterial physiology, identify new potential drug targets, and elucidate key aspects of phage-bacterial dynamics.

## Materials and methods

### Growth of mycobacteria and mycobacteriophages

*Mycobacterium smegmatis* mc^2^155 was grown at 37 °C in either Middlebrook 7H9 (Difco) with the addition of 10% ADC (12% Dextrose, 5.1% NaCl_2_, 30% Albumin), 50 ng/µl carbenicillin (CB), 10 ng/µl cycloheximide (CHX), and 0.05% Tween 80 for liquid growth, or in Middlebrook 7H10 (Difco) with the addition of 10% ADC, 50 ng/µl carbenicillin (CB) and 10 ng/µl cycloheximide (CHX) for solid media. When the culture was used for phage infections, 1 mM CaCl_2_ was added to liquid and solid media, and Tween 80 was not added. Typically, it takes three days for visible colonies to form on solid media.

Propagation of phages was done by infecting 500 µl of a saturated *M. smegmatis* culture with 10 µl phage lysate for 10 min at room temperature, adding 2.25 mls of Middlebrook Top Agar (MBTA; Middlebrook 7H9, 1 mM CaCl_2_) pouring onto solid media, and incubating at 37 °C for one to two days for plaque formation.

### Plasmid construction

The vector pTNds is a hygromycin (Hyg) resistant, Tet-ON Gateway Cloning destination plasmid, that replicates extrachromosomally in *M. smegmatis* mc^2^155^31^. The vector pTNdi is also a Tet-ON Gateway Cloning destination plasmid but is streptomycin (STR) resistant and integration proficient. Both vectors have been described previously^31^. Cloning of genes into pTNds or pTNdi was done according to Invitrogen’s ‘Gateway Technology with Clonase II’ user manual but with reduced reaction volume. Due to the nature of the Gateway Cloning technique and the location of the Gateway Cloning Cassette, a ribosomal binding site (RBS) must be included with the gene to be expressed, hence the inclusion of 30 bp DNA upstream of each gene. Protein overexpression from the extrachromosomal plasmids was induced by adding 100 ng/ml anhydrotetracycline (ATc) to the growth media for extrachromosomal plasmids, and 300 ng/ml ATc for integrated plasmids.

### Identification of toxic/inhibitory mycobacteriophage-encoded genes in *M. smegmatis*

Approximately 50 ng of each plasmid was transformed into 100 µl M*. smegmatis* mc^2^155 electrocompetent cells with 2.5 kV, 1,000 Ω, and capacitance at 25 µF as described previously^48^. After recovery for 2–4 h in liquid medium, transformants were recovered on selective media. Individual transformants were picked and patched onto solid media lacking or containing 100 ng/ml ATc (300 ng/ml ATc for pTNdi-based vectors) and incubated at 37 °C for 2–3 days. As a gene expression control, the pTNds or pTNdi with mCherry was also transformed and patched. Three days later, the patches with mCherry on the induced plate were visibly pink.

Genes were analyzed for potential functions using a variety of bioinformatic strategies, including psiBLAST and HHpred^49,50^.

### Toxicity Index assignment

A Toxicity Index (TI) value of 1–5 was assigned for each construct based on growth of bacterial patches on solid media in the presence of inducer, as follows: 5, little or no bacterial growth, 4, some but very little growth, 3, growth on some patches, but somewhat variable for different patches, 2, evident although not good bacterial growth, 1, good bacterial growth but slightly smaller patches than in the absence of inducer. No difference in growth was assigned a TI of zero. Although the assignment of TI is based on the above characteristics, it is also somewhat objective and based on relative ranking of toxicity with other strains assayed similarly.

### Live/dead staining

*M. smegmatis* with pTNds vector carrying Wildcat *11* was grown in liquid culture at 37 °C with shaking till OD_600_ is ~ 0.5. The culture was then induced with 100 ng/ml ATc for six hours at 37 °C with shaking. The induced cells were treated with Live/Dead BacLight Bacterial Viability Kit for microscopy (ThermoFisher) following the recommended protocol in the kit.

### Microscopy

Light microscopy was done using an Axiostar plus Transmitted-Light Microscopy (Zeiss; https://www.micro-shop.zeiss.com/en/us/system/axiovision+software-software+axiovision-software/6014/) with 100X objective lenses, and images were acquired using AxioCam MRc5 camera and AxioVision software (version 4.6.3.0). To prepare a microscope sample slide, a 5–10 µl liquid culture was pipetted onto a slide. Then a coverslip was placed on top of the drop, followed by hard pressing the coverslip with a piece of Kimwipe to absorb excess liquid. The coverslip was then sealed with nail polish. Fluorescence microscopy was performed using the same microscope with the attachment of HBO 50 Microscope Illuminator (Zeiss) and filter 42002 (Chroma).

### Co-immunoprecipitation

The co-IP protocol is similar to the one described previously^31^. In brief, a 100 ml liquid culture of *M. smegmatis* containing the desired plasmid was grown to OD_600_ ~ 0.5 at 37 °C at 250 rpm. Protein expression was induced for 4 to 7 h, and cell pellets were isolated and resuspended in 500 µl lysis buffer (50 mM Tris–HCl, 1 mM EDTA, 0.5% Triton X-100, 1 mM PMSF) followed by sonication and centrifugation to collect the lysates; 4,000 µg to 8,000 µg total protein of each lysate was used for co-IP with Pierce HA tag IP/Co-IP kit with anti-HA agarose beads or anti-HA magnetic Beads (ThermoFisher), washed with lysis buffer and TBS (25 mM Tris–HCl, 150 mM NaCl, pH7.2–7.4), and 2X Non-reducing sample buffer was used for elution with β-mercaptoethanol added after elution. The eluate was analyzed on a 4–20% SDS-PAGE gradient gel and imaged using GelDoc XR + (Bio-Rad) with ImageLab (Bio-Rad, version 3.0, build 11, https://www.bio-rad.com/en-us/product/image-lab-software?ID=KRE6P5E8Z). The eluates or excised candidate bands were trypsin digested, and identified by Biomedical Mass Spectrometry Center at the University of Pittsburgh with nano-reverse phase HPLC interfaced with an LTQ linear ion trap mass spectrometry along with SEQUEST search engine and Scaffold software for statistical validation, or identified by University of California at Davis Proteomics Core Facility. For Wildcat gp11, Pierce anti-HA magnetic beads were used (ThermoFisher). After the washing steps, protein-bound magnetic beads were not eluted and were directly digested on beads and analyzed by tandem Mass Spectrometry with X! Tandem search engine by the University of California at Davis Proteomics Core Facility.

### RNA sequencing analysis

Mycobacterial liquid cultures of OD_600_ ~ 0.7 were infected with Wildcat phage lysate at a multiplicity of infection of three for 10 min before incubating at 37 °C. Thirty minutes and 150 min post-infection, 4 ml samples were collected for RNA isolation using matrix lysing tubes (MP Biomedicals) and RNeasy Mini Kit (Qiagen). rRNA was then removed using RiboZero rRNA removal kit (Gram-positive bacteria, Illumina). RNAseq was then performed and analyzed as described previously^11^. RNAseq data was visualized using Integrative Genomics Viewer (version 2.3.92)^51^ combined with Phamerator-generated genome maps^52^.

## Supplementary information

Supplementary Information.

## Data Availability

The data generated or analysed during this study are included in this published article (and its Supplementary Information files). Phage genome sequence information is available in GenBank and at https://phagesdb.org. RNAseq data of Wildcat is deposited at Gene Expression Omnibus (https://www.ncbi.nlm.nih.gov/geo/) with accession number GSE149160.
