# Supplementary material for: Identification of mycobacteriophage toxic genes reveals new features of mycobacterial physiology and morphology
Source: Sci Rep. 2020 Sep 4;10:14670. doi: 10.1038/s41598-020-71588-5 (PMC7474061; doi:10.1038/s41598-020-71588-5)
Supplement: Supplementary file 1 — Supplementary Information. [file 41598_2020_71588_MOESM1_ESM.pdf]

Supplementary Information

Identification of mycobacteriophage toxic genes reveals new features of mycobacterial physiology and morphology

Running title: Mycobacteriophage toxic genes

Ching-Chung Ko and Graham F. Hatfull*

Department of Biological Sciences
University of Pittsburgh
Pittsburgh, PA 15260

*Corresponding author: gfh@pitt.edu

Table S1. Mycobacteriophage genes screened for toxicity.

Supplementary Figure Legends

Figure S1. Toxic mycobacteriophage-encoded genes: Toxicity Index = 5. Five individual *M. smegmatis* transformants carrying plasmids expressing mycobacteriophage genes were patched onto solid media lacking (-ATc) or containing (+ATc) inducer. All genes were inserted into an extrachromosomally-replicating vector with the exception of LHTSCC gene *91* which was inserted into an integration-proficient vector. Plates were incubated for 2-3 days at 37° C. The toxicity index (TI) is shown at the right, where 0 represents no toxicity, and 5 corresponds to the strongest toxicity.

Figure S2. Toxic mycobacteriophage-encoded genes: Toxicity Index = 1-4. Five individual *M. smegmatis* transformants carrying plasmids expressing mycobacteriophage genes were patched onto solid media lacking (-ATc) or containing (+ATc) inducer. Plates were incubated for 2-3 days at 37° C. The toxicity index (TI) is shown at the right, where 0 represents no toxicity, and 5 corresponds to the strongest toxicity.

Figure S3. Impact of toxic gene expression of cellular morphology. Liquid cultures of *M. smegmatis* transformants carrying mycobacteriophage toxic genes were induced by addition of ATc at time 0, and examined by bright field microscopy after 0, 4, 8 and 12 hours. 5 μm scale bars are shown. Images for Troll *60*, Troll *72,* Troll *85*, Wildcat *144*, Wildcat *166*, and LHTSCC *46* are shown.

Figure S4. Impact of toxic gene expression of cellular morphology. Liquid cultures of *M. smegmatis* transformants carrying mycobacteriophage toxic genes were induced by addition of ATc at time 0, and examined by bright field microscopy after 0, 4, 8 and 12 hours. 5 μm scale bars are shown. Images for LHTSCC *49*, Wildcat *168*, Wildcat *11*, Wildcat *165*, Wildcat *145* and Hammer *103* are shown.

Figure S5. Impact of toxic gene expression of cellular morphology. Liquid cultures of *M. smegmatis* transformants carrying mycobacteriophage toxic genes were induced by addition of ATc at time 0, and examined by bright field microscopy after 0, 4, 8 and 12 hours. 5 μm scale bars are shown. Images for Konstantine *66*, LHTSCC *48*, LHTSCC *83*, Wildcat *13*, Wildcat *143* and the empty vector are shown.

Figure S6. Impact of toxic gene expression of cellular morphology. Liquid cultures of *M. smegmatis* transformants carrying mycobacteriophage toxic genes were induced by addition of ATc at time 0, and examined by bright field microscopy after 0, 4, 8 and 12 hours. 5 μm scale bars are shown. Images for BAKA *17*, LHTSCC *91*, Rosebush *44* and Wildcat *163* are shown.

Figure S7. Toxicity of C-terminally eGFP-tagged toxic proteins. Mycobacteriophage toxic proteins were C-terminally fused to eGFP and tested for toxicity when induced with ATc. Five *M. smegmatis* transformants were patched onto solid media with or without ATc to test for toxicity. The Toxicity Index (TI) is shown at the right.

Figure S8. Conserved sequence motifs in phage Wildcat. Conserved motifs were identified in the Wildcat genome within the leftwards transcribed set of genes from *143* to *171* (see Fig. 7). The two motifs (Motif 1 and Motif 2) are present between the genes shown to right of the aligned sequences, and the coordinates are indicated. The sequence shown corresponds to the bottom DNA strand of the genome represented in Fig. 7.

Figure S9. Raw uncropped images of data shown in Figure 8. Images are labeled as indicated in Figure 8.

Table S1. Mycobacteriophage genes screened for toxicity.

Cluster	Phage	Gene	aa	Pham[1]	Start codon	Features	TI[2]
A1	Solon	*82*	51	26857	ATG	NKF	0
		83	68	112878	ATG	NKF	0
		84	54	96015	ATG	NKF	0
		86	156	92685	ATG	NKF	0
A2	Redrock	*38*	89	114213	GTG	ParB	0
A4	LHTSCC	*1*	100	113528	GTG	HNH domain	0
		17	104	33864	ATG	NKF	0
		36[3]	57	111670	ATG	NKF	0
		38	52	78860	ATG	NKF	0
		39	52	113773	ATG	Lipoprotein	1
		40[3]	88	113352	ATG	NKF	0
		42[3]	118	95528	GTG	NKF	0
		43	91	23422	ATG	NKF	0
		44	68	113026	ATG	NKF	0
		45[3]	76	109361	GTG	NKF	0
		46	56	15373	ATG	NKF	5
		48	100	112786	ATG	DNA binding	5
		49	57	100472	ATG	Lipoprotein	5
		55	91	115372	ATG	NKF	0
		56	25	113590	ATG	NKF	2
		61[3]	28	113148	GTG	NKF	0
		63[3]	126	113354	GTG	NKF	0
		67	26	8660	GTG	NKF	0
		68	64	113262	GTG	NKF	0
		69	74	114216	ATG	NKF	0
		71	140	112597	GTG	NKF	0
		73[3]	44	112896	TTG	NKF	0
		76	46	101126	ATG	NKF	2
		78	52	80151	ATG	NKF	0
		79[3]	47	106215	ATG	NKF	0
		82	56	113221	ATG	NKF	0
		83	156	112954	ATG	SprT-like	5
		86	23	29311	ATG	NKF	0
		87	29	35764	ATG	NKF	0
		88	70	114427	GTG	NKF	0
		89	68	7915	ATG	NKF	4
		91	39	11360	ATG	NKF	5
A6	Hammer	*103*	26	113384	GTG	NKF	5
		104	109	113225	ATG	NKF	0
		107	76	32170	ATG	NKF	0
B2	Rosebush	*22*	37	20964	ATG	NKF	0
		23	133	31146	ATG	NKF	0
		43	59	115118	GTG	DNA binding	0
		44	129	114195	ATG	DUF4447	5
		61	89	114869	GTG	NKF	0
		62	233	112702	ATG	NKF	0
		64	113	10356	GTG	NKF	0
		70	74	114699	GTG	NKF	3
		73[3]	102	6468	ATG	NKF	0
		74	103	112057	ATG	NKF	0
		75	81	112586	GTG	NKF	3
		76	105	92050	TTG	NKF	0

		78	53	93825	ATG	NKF	0
		79	70	45208	ATG	NKF	0
B3	Kamiyu	*22*	36	92069	ATG	NKF	0
		60	45	11706	GTG	NKF	0
		63	39	82987	GTG	NKF	1
		81	35	92829	GTG	NKF	0
		88	62	1941	ATG	NKF	0
		89	64	112612	ATG	NKF	0
		90	51	79882	ATG	NKF	0
		91	119	8630	ATG	NKF	0
		92	70	19333	ATG	NKF	0
		93	127	98470	GTG	NKF	0
D1	Troll4	*48*	34	113169	GTG	NKF	0
		49	102	113743	ATG	NKF	0
		52	71	3438	ATG	NKF	0
		58	62	83102	GTG	NKF	0
		60	302	114288	ATG	DUF669	5
		63	176	92443	ATG	Methyltransferase	4
		65	236	32751	GTG	FabG/L-xylulose reductase	0
		69	58	6789	GTG	NKF	0
		72	117	79617	GTG	NKF	5
		75	71	2163	ATG	DNA binding	4
		76	146	101315	ATG	DUF732	4
		81	86	4693	ATG	NKF	0
		85	179	95223	ATG	NKF	5
		87	37	6853	ATG	NKF	0
E	Bask21	*31*	94	107400	ATG	NKF	0
		34	112	112654	GTG	NKF	0
F1	Fruitloop	*25*	65	113578	GTG	NKF	0
		26	94	107400	ATG	NKF	0
		48	87	114426	ATG	NKF	0
		49	49	115117	ATG	NKF	0
		51	223	114366	GTG	NKF	0
		53	62	13950	ATG	NKF	0
		56	166	18199	ATG	NKF	0
		57	121	114409	ATG	DNA binding	3
		59[3]	81	96291	ATG	NKF	0
		60	192	13399	ATG	Rad52-like	2
		65	190	94377	ATG	NKF	0
		66	90	81676	ATG	NKF	0
		68	63	114010	ATG	NKF	0
		71	110	95635	ATG	MPME2	0
		72[3]	67	1311	GTG	NKF	0
		73	59	80603	ATG	NKF	0
		74	123	114526	ATG	NKF	0
		76	116	115073	ATG	NKF	0
		77	49	22693	ATG	NKF	0
		78	94	115427	ATG	NKF	2
		79	92	114883	ATG	NKF	0
		83	139	94176	ATG	NKF	4
		84	187	114309	GTG	NKF	0
		86	88	113732	TTG	NKF	0
		87	87	20591	ATG	NKF	0
		88	59	94824	ATG	NKF	0
		89	38	113968	ATG	NKF	0
		90	112	82909	ATG	NKF	0

		92	74	112637	ATG	NKF	0
		93	55	110512	GTG	NKF	0
		94	61	114664	ATG	NKF	1
		95	50	13554	ATG	NKF	0
		96	137	115058	ATG	NKF	0
		100	152	111242	ATG	NKF	0
		101	81	82003	ATG	NKF	0
H1	Konstantine	*66*	89	14428	ATG	NKF	5
		67	123	91373	ATG	NKF	0
		69	42	5851	ATG	NKF	0
I1	Babsiella	*23*	49	2296	GTG	NKF	0
		31	71	28838	ATG	HicA-like toxin	0
		50	354	7343	GTG	FtsK domain	4
		51	168	21275	ATG	RusA-like resolvase	0
		55	102	19001	TTG	DNA methylase domain	0
		68	47	33350	ATG	NKF	4
J1	BAKA	*17*	186	113249	TTG	M34 peptidase-like	5
		18	100	22540	ATG	NKF	0
		19	47	92692	ATG	NKF	0
		211	60	19647	ATG	NKF	0
		212	58	17215	ATG	NKF	0
		213	56	95888	GTG	NKF	0
		214	86	8736	ATG	NKF	0
		215	76	16641	ATG	NKF	0
		216	82	10626	ATG	NKF	0
		217	170	96194	ATG	NKF	0
		218	97	109355	GTG	DNA binding	0
		219	169	21277	ATG	NKF	0
		224	50	12685	ATG	NKF	2
		225	48	7693	ATG	NKF	0
		234	27	6824	ATG	NKF	0
		235	33	52485	GTG	NKF	0
		236	66	96289	ATG	NKF	0
		237	69	93617	TTG	NKF	0
		238	44	79616	ATG	NKF	0
		239	124	10580	ATG	NKF	0
V	Wildcat	*1*[3]	71	3435	ATG	NKF	0
		4	38	4749	ATG	NKF	0
		5[3]	66	52228	ATG	NKF	0
		10	77	3798	ATG	NKF	0
		11	215	94450	ATG	Methyltransferase	5
		13	77	3799	ATG	LexA-like	5
		14[3]	49	15915	ATG	NKF	0
		15[3]	104	10662	ATG	NKF	0
		16	49	15920	ATG	NKF	0
		18[3]	100	52416	ATG	NKF	0
		20	84	6874	ATG	NKF	0
		21	87	16363	ATG	NKF	0
		22	124	23375	ATG	NKF	4
		23	109	20820	GTG	NKF	0
		101	74	16123	ATG	NKF	0
		106	57	9154	ATG	NKF	0
		109	28	20045	ATG	NKF	0
		125	120	17030	GTG	NKF	0
		129	81	13329	ATG	NKF	0
		131	44	16582	GTG	NKF	0

143	59	113004	ATG	NKF	5
144	185	1117	ATG	NKF	5
145	55	96213	ATG	NKF	5
146	89	94423	ATG	NKF	0
147	92	113634	ATG	NKF	5
148[3]	109	92627	ATG	NKF	0
149	65	14768	GTG	NKF	0
150	44	38932	ATG	NKF	0
151	78	2268	ATG	NKF	0
152	112	114532	ATG	NKF	0
153[3]	71	28761	ATG	NKF	0
154	84	11186	ATG	NKF	0
155	134	12316	ATG	NKF	2
156	74	12497	ATG	NKF	0
157[3]	69	12950	TTG	NKF	0
158[3]	110	4997	ATG	NKF	0
159	105	4122	TTG	NKF	2
160	89	16167	GTG	NKF	0
161	96	9914	ATG	NKF	0
162	117	93782	GTG	NKF	4
163	121	114092	ATG	NKF	5
164	106	104746	ATG	NKF	0
165	91	23421	ATG	NKF	5
166	128	95405	GTG	NKF	5
167	121	94272	GTG	NKF	1
168	97	94773	ATG	NKF	5
169	60	11887	ATG	NKF	0
170	70	114773	ATG	NKF	0
171	82	94194	GTG	NKF	0

[1]Pham designation as of 2-21-2020 at https://phagesdb.org.

[2]Toxicity Index (TI) scored from 1 to 5, with 5 being the most toxic and 1 being the least toxic; TI = 0 represents no toxicity.

[3]Plasmid clone contains 1-3 base substitutions in the coding region introduced in PCR amplification and cloning that are unlikely to influence any toxic activity.

Table S2. Mass spectrometry analysis of co-immunoprecipitated proteins

Toxic Protein	Band # / Whole Eluate Analyzed[1]	Target Identified[2]	Molecular Weight (kD)	Target Function	Target Total Spectrum Count[3]	
					Co-IP with wild type toxic protein	Co-IP with HA-tagged toxic protein
LHTSCC gp46	B2	Msmeg_6227	27	PadR family protein	1	124
		thiD (Msmeg_0825)	29	Phosphomethylpyrimidine kinase	9	20
		phoP (Msmeg_5872)	27	DNA-binding response regulator	11	18
		rpsE (Msmeg_1472)	22	30S ribosomal protein S5	16	16
		hisF (Msmeg_3211)	27	Imidazole gylcerol phosphate synthase subunit HisF	7	14
	B3	tuf (Msmeg_1401)	44	Elongation factor Tu	78	201
		Msmeg_4328	44	3-oxoacyl-[acyl-carrier-protein] synthase 2	15	58
		Msmeg_4327	44	3-oxoacyl-[acyl-carrier-protein] synthase 1	24	42
		hemL_2	47	Glutamate-1-semialdehyde 2,1-aminomutase	23	39
		Msmeg_3513	44	Uncharacterized N-acetyltransferase	19	38
	B4	rpoC (Msmeg_1368)	147	DNA-directed RNA polymerase subunit beta'	128	409
		Msmeg_4757	330	Fatty acid synthase	29	61
		carB (Msmeg_3047)	120	Carbamoyl-phosphate synthase large chain	44	60
		kgd (Msmeg_5049)	136	Multifunctional 2-oxoglutarate metabolism enzyme	51	51
		smc (Msmeg_2423)	130	Chromosome partition protein	13	39
	eluate	Msmeg_4757	330	Fatty acid synthase	23	156

		rpoC (Msmeg_1368)	147	DNA-directed RNA polymerase subunit beta'	22	130
		rpoB (Msmeg_1367)	129	DNA-directed RNA polymerase subunit beta	18	87
		tuf (Msmeg_1401)	44	Elongation factor Tu	25	78
		mps	646	Peptide synthetase	0	73
		Msmeg_0408	390	Type I modular polyketide synthase	0	57
		Msmeg_6227	27	PadR family protein	0	53
		pks13 (Msmei_6224)	197	Polyketide synthase	4	49
		glpK (Msmeg_6759)	55	Glycerol kinase	15	40
		dnaK (Msmeg_0709)	67	Chaperone protein	27	38
		LHTSCC gp46	7		0	11
LHTSCC gp49	B2	rpoC (Msmeg_1368)	147	DNA-directed RNA polymerase subunit beta'	96	185
		kgd (Msmeg_5049)	136	Multifunctional 2-oxoglutarate metabolism enzyme	10	14
		pks13 (Msmei_6224)	197	Polyketide synthase	0	11
		Msmeg_4757	330	Fatty acid synthase	0	9
		hrpA (Msmeg_6587)	146	ATP-dependent helicase	3	5
LHTSCC gp83	B1	rplP (Msmeg_1443)	16	Ribosomal protein L16	4	41
		rpsK (Msmeg_1522)	15	Ribosomal protein S11	2	28
		rpsG (Msmeg_1399)	18	Ribosomal protein S7	14	26
		Msmeg_4272	12	HesB/YadR/YfhF family protein	12	23
		Msmeg_0238	17	O-acetylhomoserine/O-acetylserine sulfhydrylase	2	20
	B2	LHTSCC gp83	19		4	32
	B3	rplC (Msmeg_1436)	23	Ribosomal protein L3	10	37

		Msmeg_1046, Msmeg_2326	31	ABC-type molybdenum transport system, ATPase component	12	31
		Msmei_4292	28	Isochorismatase hydrolase	16	26
		Msmeg_6385	27	Short chain dehydrogenase	17	26
		thiD (Msmeg_0825)	29	Phosphomethylpyrimidine kinase	15	24
	B4	rpoC (Msmeg_1368)	147	DNA-directed RNA polymerase subunit beta'	151	294
		Msmeg_4757	330	Fatty acid synthase	81	147
		rpoB (Msmeg_1367)	128	DNA-directed RNA polymerase subunit beta	100	142
		mps	646	Peptide synthetase	49	127
		pks13 (Msmei_6224)	197	Polyketide synthase	52	118
Wildcat gp11	Eluate[4]	Msmeg_4757	330	Fatty acid synthase	189	179
		glpK (Msmeg_6759)	55	Glycerol kinase	112	156
		rpoC (Msmeg_1368)	147	DNA-directed RNA polymerase subunit beta'	124	145
		pks13 (Msmei_6224)	197	Polyketide synthase	90	125
		Msmeg_6227	27	PadR family protein	0	119
		mps	646	Peptide synthetase	62	113
		ilvC (Msmeg_2374)	36	Ketol-acid reductoisomerase (NADP(+))	76	98
		Msmeg_4328	44	3-oxoacyl-[acyl-carrier-protein] synthase 2	125	95
		tuf (Msmeg_1401)	44	Elongation factor Tu	89	89
		ino1 (Msmeg_6904)	39	Inositol-3-phosphate synthase	73	88
		Wildcat gp11	26		17	172
Wildcat gp13	B2	Msmeg_1285	88	Tetratricopeptide repeat family protein	26	121
		clpC1 (Msmeg_6091)	94	ATP-dependent Clp protease ATP-binding subunit	59	73

		rho (Msmeg_4954)	72	transcription termination factor	61	72
		Msmeg_4757	330	Fatty acid synthase	40	70
		mps	646	Peptide synthetase	27	50
Wildcat gp144	eluate-in gel digest	groL1 (Msmeg_0880)	56	Chaperonin GroL	0	25
		Msmeg_1807	63	Acetyl-/propionyl-coenzyme A carboxylase alpha chain	0	25
		valS (Msmeg_4630)	98	Valine-tRNA synthetase	0	21
		rpoC (Msmeg_1368)	147	DNA-directed RNA polymerase subunit beta'	0	20
		dnaK (Msmeg_0709)	67	Chaperone protein	0	17
		tuf (Msmeg_1401)	44	Elongation factor Tu	0	13
		Msmeg_4709	26	Enoyl-CoA hydratase	0	13
		folD (Msmeg_1647)	30	Bifunctional protein: Methylenetetrahydrofolate dehydrogenase and Methenyltetrahydrofolate cyclohydrolase	0	11
		rpsA (Msmeg_3833)	53	30S ribosomal protein S1	0	11
		sdhA (Msmeg_1670)	64	Succinate dehydrogenase flavoprotein subunit	0	11
		Wildcat gp144	22		0	10
	eluate-in solution digest	dnaK (Msmeg_0709)	67	Chaperone protein	0	35
		valS (Msmeg_4630)	98	Valine-tRNA synthetase	0	34
		groL1 (Msmeg_0880)	56	Chaperonin GroL	0	33
		rpoB (Msmeg_1367)	129	DNA-directed RNA polymerase subunit beta	0	33
		Msmeg_1807	63	Acetyl-/propionyl-coenzyme A carboxylase alpha chain	0	31
		rpoC (Msmeg_1368)	147	DNA-directed RNA polymerase subunit beta'	0	28
		Msmeg_1654	83	Isocitrate dehydrogenase, NADP-dependent	0	25

		Msmeg_4757	330	Fatty acid synthase	0	22
		groL2 (Msmeg_1583)	56	Chaperonin GroL	0	22
		rpsA (Msmeg_3833)	53	30S ribosomal protein S1	0	22
		Wildcat gp144	22		0	31
Wildcat gp145	B2	Msmei_1480 / Msmeg_1516	61	Thioredoxin-disulfide reductase	33	290
		groL2 (Msmeg_1583)	56	Chaperonin GroL	46	41
		serA (Msmeg_2378)	54	D-3-phosphoglycerate dehydrogenase	45	41
		groL1 (Msmeg_0880)	56	Chaperonin GroL	25	31
		glpD2 (Msmei_6578)	61	Glycerol-3-phosphate dehydrogenase	25	27
	eluate-in gel digest	Msmei_3558	22	Transcriptional regulator, MerR family	0	22
		Msmei_1480 / Msmeg_1516	61	Thioredoxin-disulfide reductase	0	11
		dnaK (Msmeg_0709)	67	Chaperone protein	0	2
		Msmeg_2526	74	Amine oxidase	2	2
		msrA (Msmeg_6477)	19	Peptide methionine sulfoxide reductase	1	2
	eluate-in solution digest	msrA (Msmeg_6477)	19	Peptide methionine sulfoxide reductase	12	12
		Msmei_3558	22	Transcriptional regulator, MerR family	0	11
		dnaK (Msmeg_0709)	67	Chaperone protein	0	8
		Msmeg_4272	12	HesB/YadR/YfhF family protein	2	8
		Msmei_1480 / Msmeg_1516	61	Thioredoxin-disulfide reductase	0	8
		greA (Msmeg_5263)	18	Transcription elongation factor GreA	0	5
		groS (Msmeg_1582)	11	Chaperonin GroS	0	5
		Msmeg_2526	74	Copper methylamine oxidase	5	5

		bfrB (Msmeg_6422)	20	Ferritin BfrB	2	5
		Msmeg_4528	26	Phosphoadenosine phosphosulfate reductase	0	5
		Wildcat gp145	7		0	12
Wildcat gp165	B2	Wildcat gp165	11		0	142
		Msmeg_3253	32	Uncharacterized protein	22	24
		Msmeg_0913	33	Methoxy mycolic acid synthase 1	20	14
		fabG (Msmeg_3150)	27	3-oxoacyl-[acyl-carrier-protein] reductase	12	10
		Msmeg_3020	30	Uncharacterized protein	12	10
	eluate-in solution digest	msrA (Msmeg_6477)	19	Peptide methionine sulfoxide reductase	4	5
		alc (Msmeg_5727)	35	Allantoicase	0	4
		Msmeg_1807	63	Acetyl-/propionyl-coenzyme A carboxylase alpha chain	0	4
		Msmeg_0234	74	Metallopeptidase	4	3
		mcs (Msmeg_4921)	17	Methylmalonyl-CoA epimerase	4	3
		Msmeg_2450	49	adenosylmethionine--8-amino-7-oxononanoate transaminase	3	3
		Msmei_1640	16	Endoribonuclease L-PSP	2	3
		garA (Msmeg_3647)	17	Glycogen accumulation regulator	6	3
		groL1 (Msmeg_0880)	56	Chaperonin GroL	0	3
		Msmeg_4272	12	HesB/YadR/YfhF family protein	3	2
		Wildcat gp165	11		0	18

[1]Individual protein bands are labeled according to Figure 8 (e.g. B1, B2 etc). Eluate-in solution digest indicates the whole eluate was proteolytic digested in solution. Eluate-in gel digest indicates the whole eluate was ran on an SDS-PAGE gel for a very short period of time, stained, the total eluate protein band excised (~1cm), and proteolytically digested.

[2]For eluate samples, proteins are listed that have the ten highest number of spectrum counts from Co-IP with HA-tagged toxic proteins along with toxic proteins, if identified. For the Wildcat gp145 eluate-in gel digest sample, only five targets are listed due to the limited number of targets identified. For individual excised band samples (for example, LHTSCC gp46 B2), the top five proteins with the highest numbers of spectra are listed. For LHTSCC gp83 B2 band, the identified protein is the toxic protein itself, and only the spectrum counts for the toxic protein is listed.

For Wildcat gp165 B2 band, Wildcat gp165 is identified as the most likely protein from the band, even though on SDS-PAGE the band migrates slower than its predicted molecular weight; an additional top four total target spectrum counts are listed. In general, protein targets are from *M. smegmatis* GenBank Accession #CP000480 (shown as Msmeg_xxxx), but for some proteins the corresponding proteins from *M. smegmatis* GenBank Accession # CP001663 are included (Msmei_xxxx) as peptides were identified corresponding to the former but not the latter, due to annotation differences.

[3]Settings are: Protein threshold at 99.9%, minimum number of peptides 2, and peptide threshold at 95%

[4]The sample for Mass Spec was obtained by directly digesting protein-bound Co-IP beads without the Co-IP elution step.

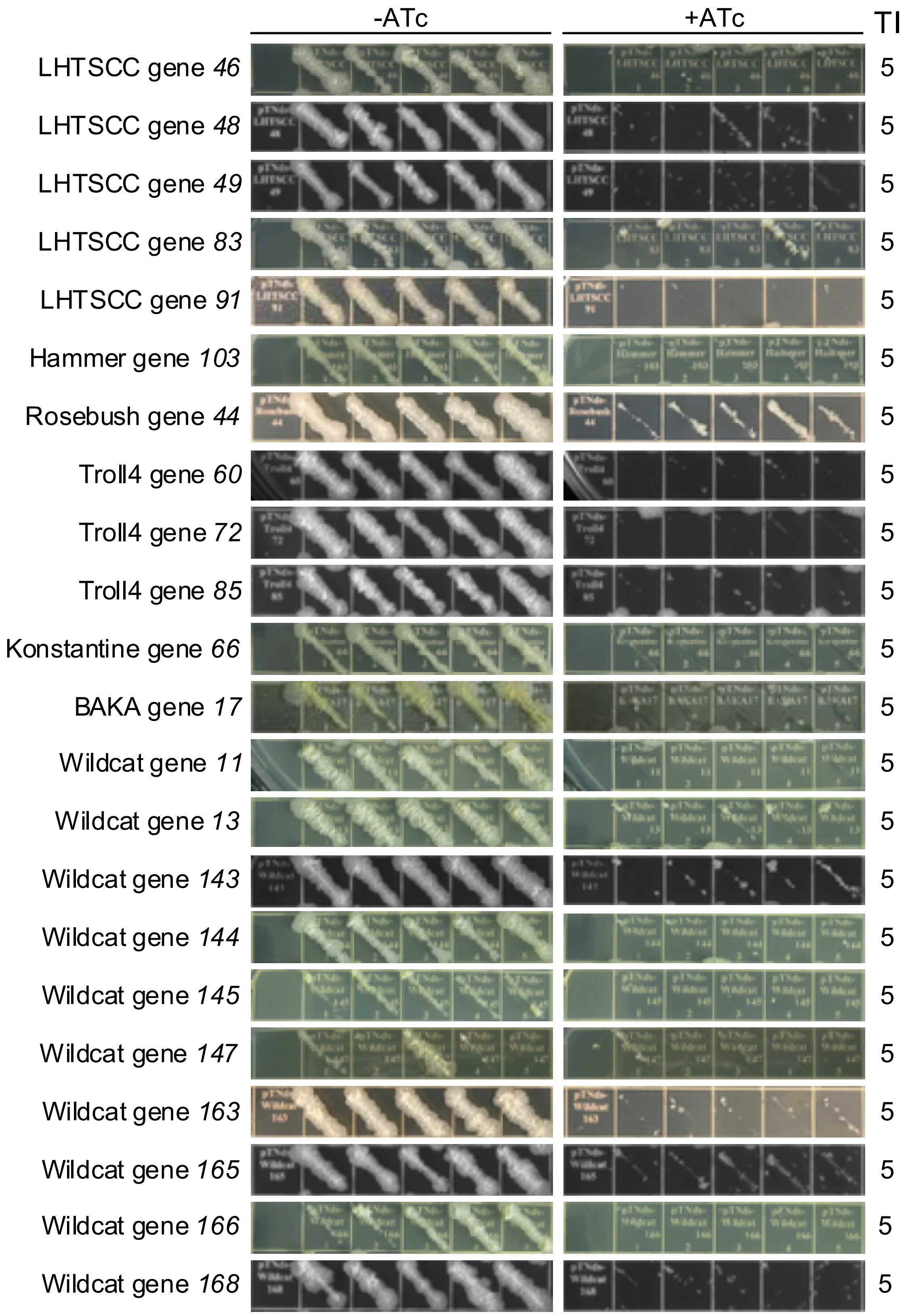
-ATc
+ATc
TI
LHTSCC gene *46* 5
LHTSCC gene *48* 5
LHTSCC gene *49* 5
LHTSCC gene *83* 5
LHTSCC gene *91* 5
Hammer gene *103* 5
Rosebush gene *44* 5
Troll4 gene *60* 5
Troll4 gene *72* 5
Troll4 gene *85* 5
Konstantine gene *66* 5
BAKA gene *17* 5
Wildcat gene *11* 5
Wildcat gene *13* 5
Wildcat gene *143* 5
Wildcat gene *144* 5
Wildcat gene *145* 5
Wildcat gene *147* 5
Wildcat gene *163* 5
Wildcat gene *165* 5
Wildcat gene *166* 5
Wildcat gene *168* 5

Figure S1

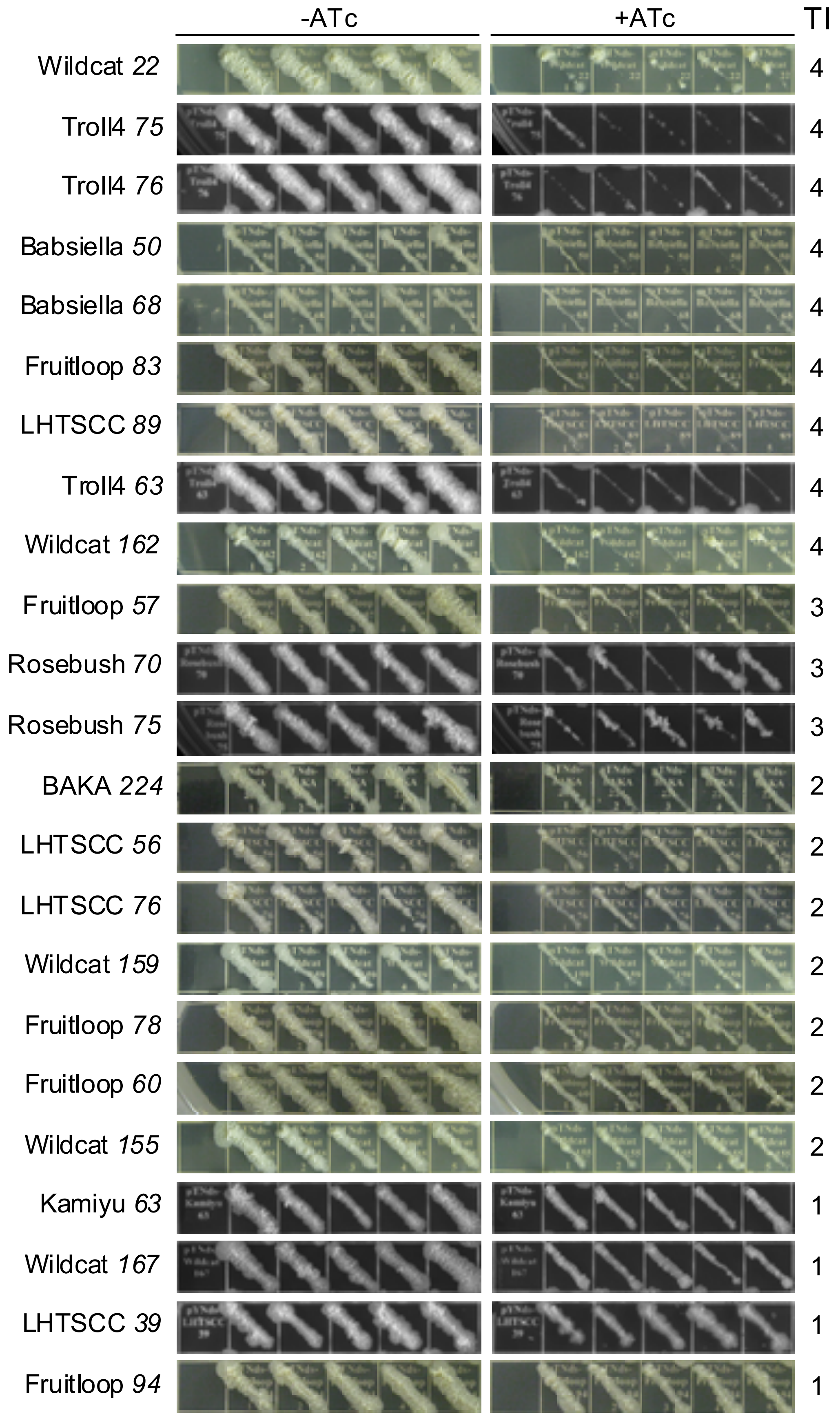
-ATc
+ATc
TI
Wildcat 22 4
Troll4 75 4
Troll4 76 4
Babsiella 50 4
Babsiella 68 4
Fruitloop 83 4
LHTSCC 89 4
Troll4 63 4
Wildcat 162 4
Fruitloop 57 3
Rosebush 70 3
Rosebush 75 3
BAKA 224 2
LHTSCC 56 2
LHTSCC 76 2
Wildcat 159 2
Fruitloop 78 2
Fruitloop 60 2
Wildcat 155 2
Kamiyu 63 1
Wildcat 167 1
LHTSCC 39 1
Fruitloop 94 1

Figure S2

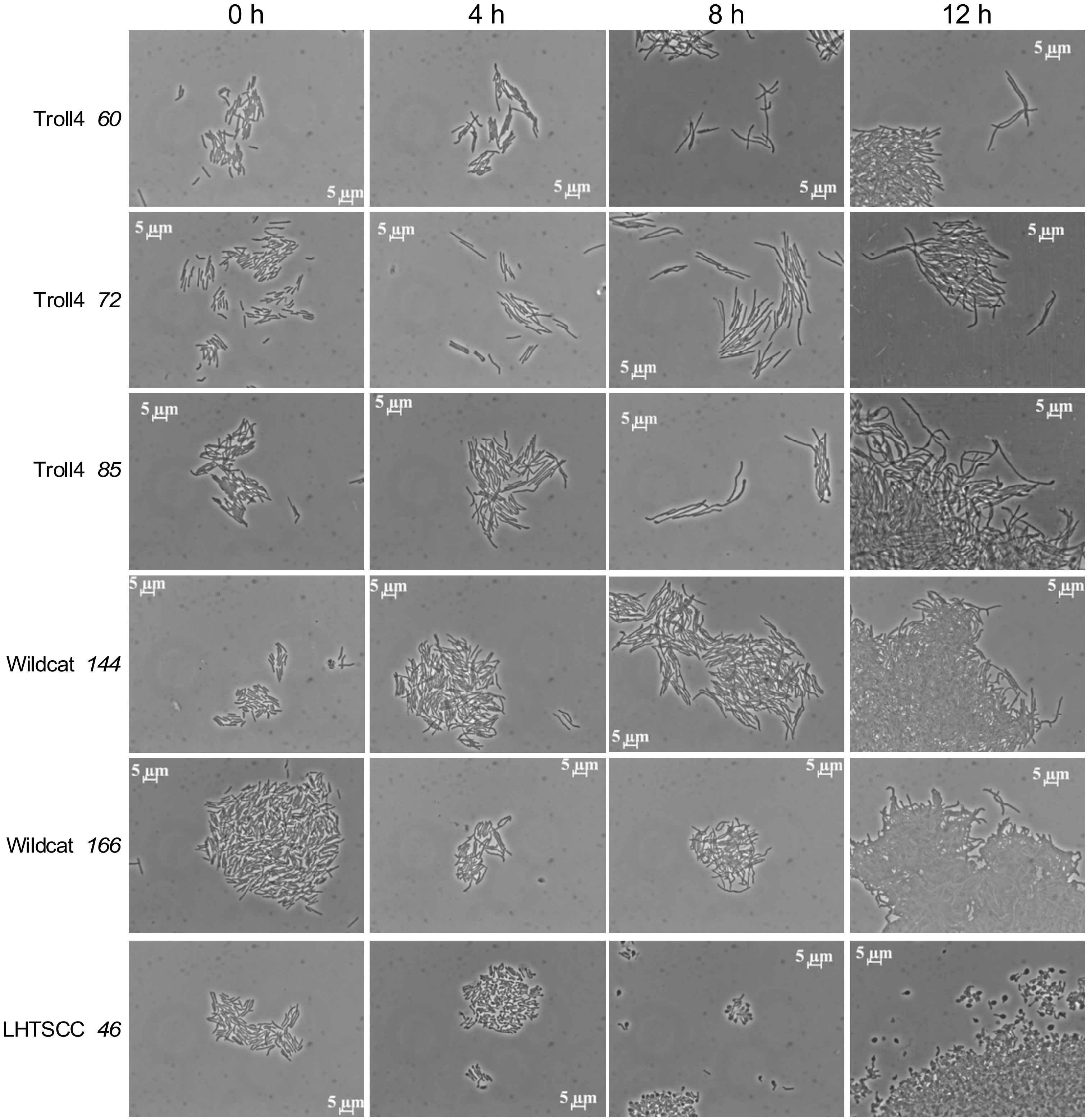

0 h
4 h
8 h
12 h
Troll4 60
Troll4 72
Troll4 85
Wildcat 144
Wildcat 166
LHTSCC 46
5 µm

Figure S3

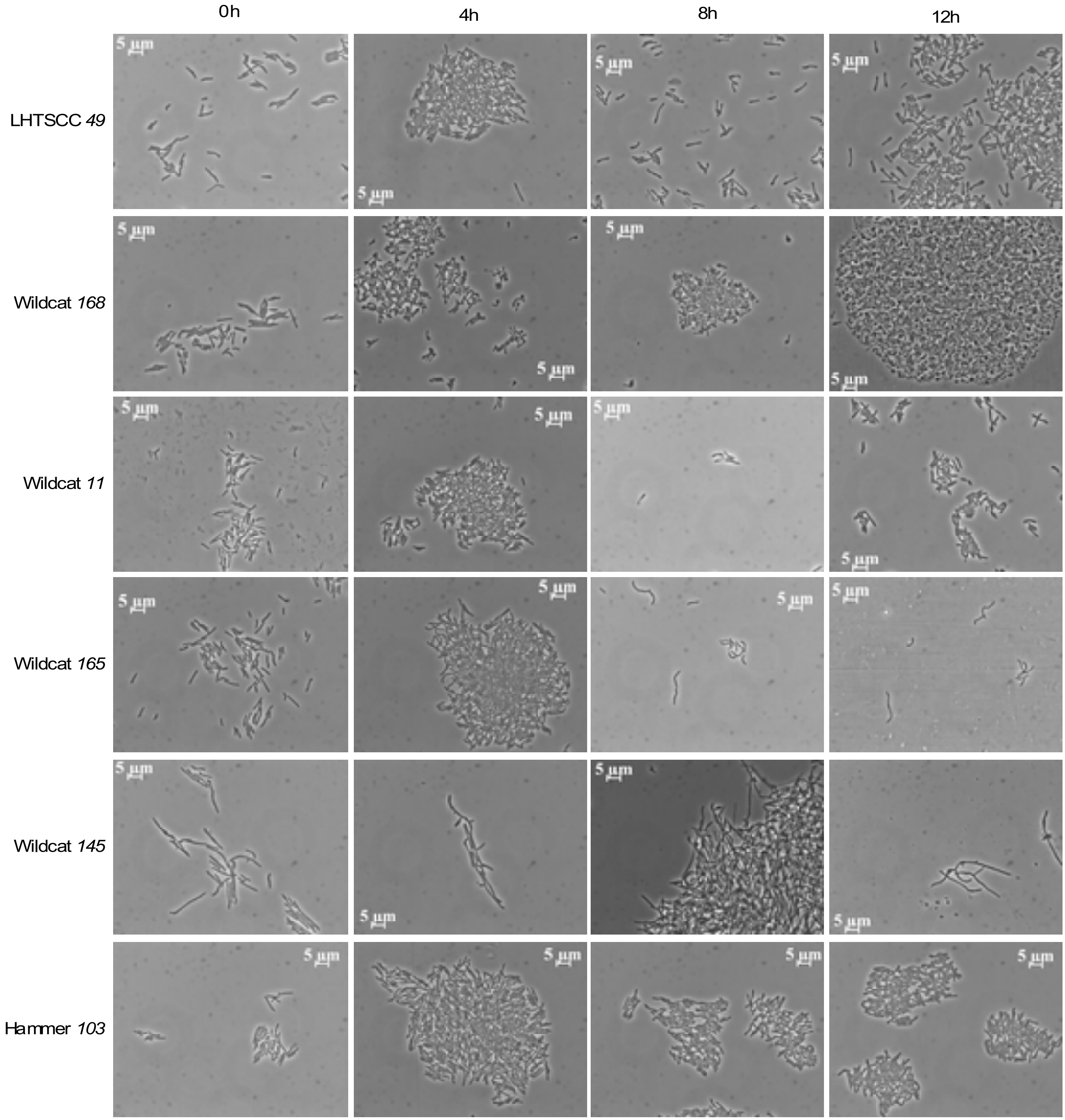
0h
4h
8h
12h
LHTSCC 49
Wildcat 168
Wildcat 11
Wildcat 165
Wildcat 145
Hammer 103
5 μm

Figure S4

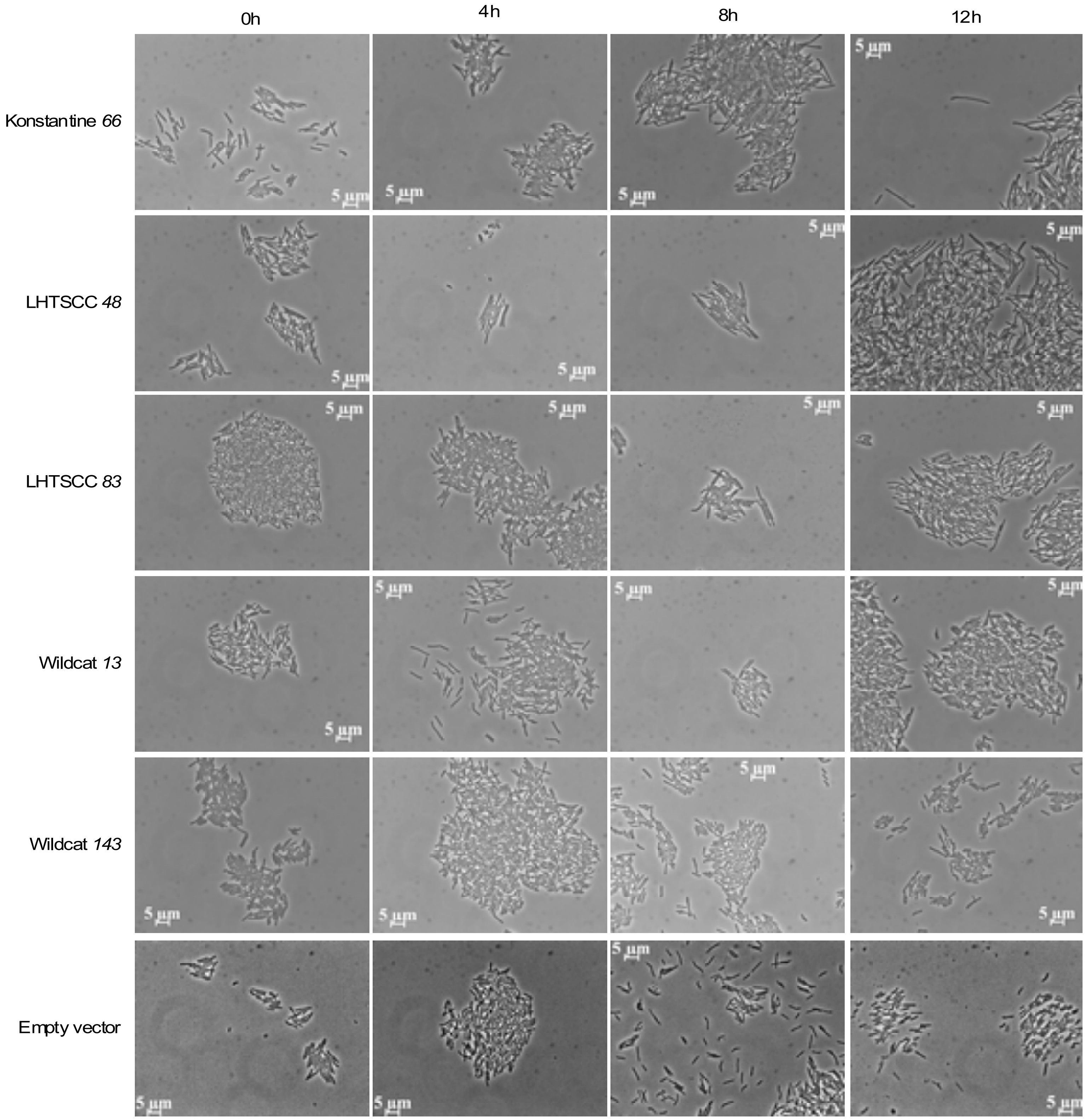
0h
4h
8h
12h
Konstantine 66
LHTSCC 48
LHTSCC 83
Wildcat 13
Wildcat 143
Empty vector
5 µm

Figure S5

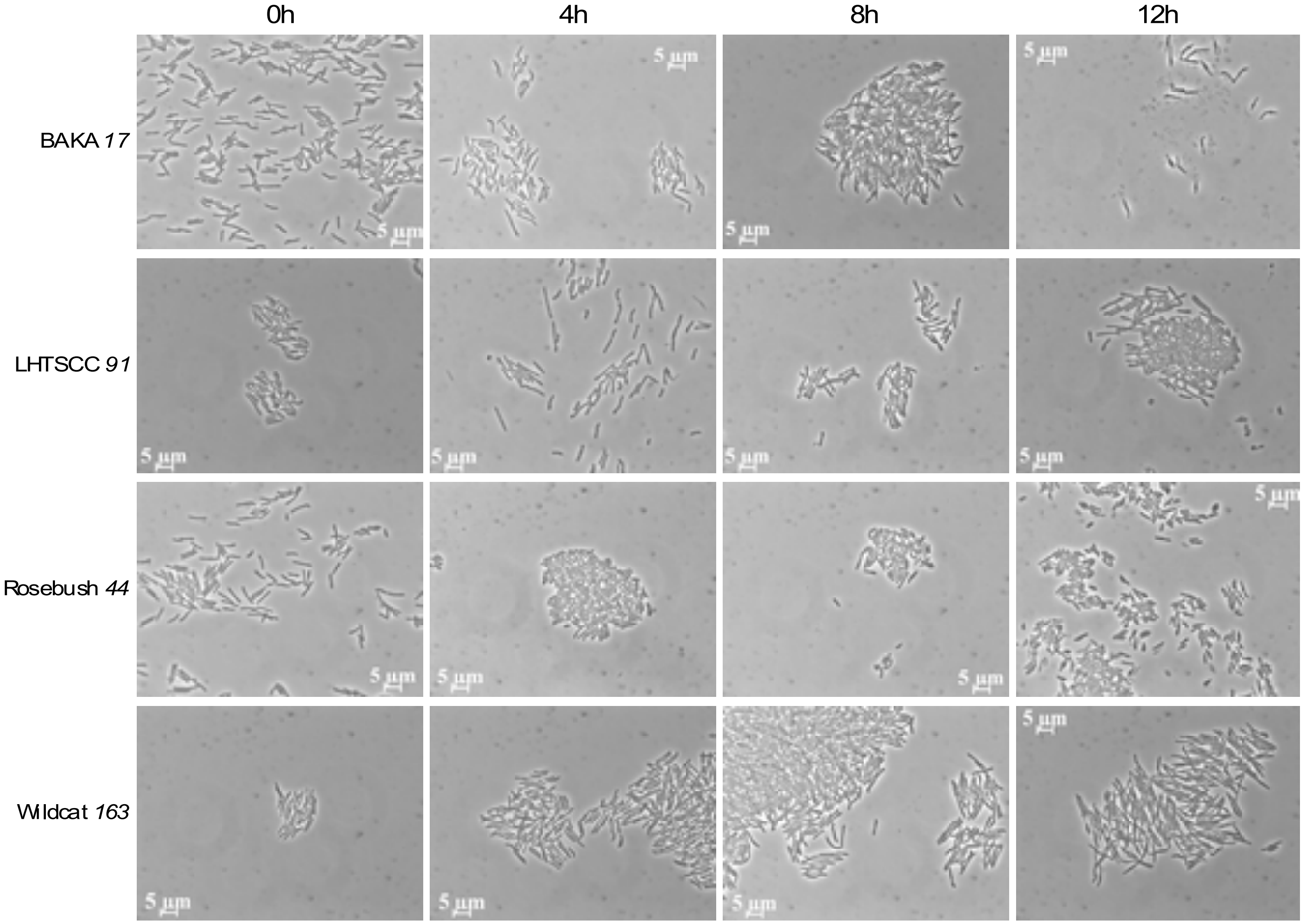

Figure S6

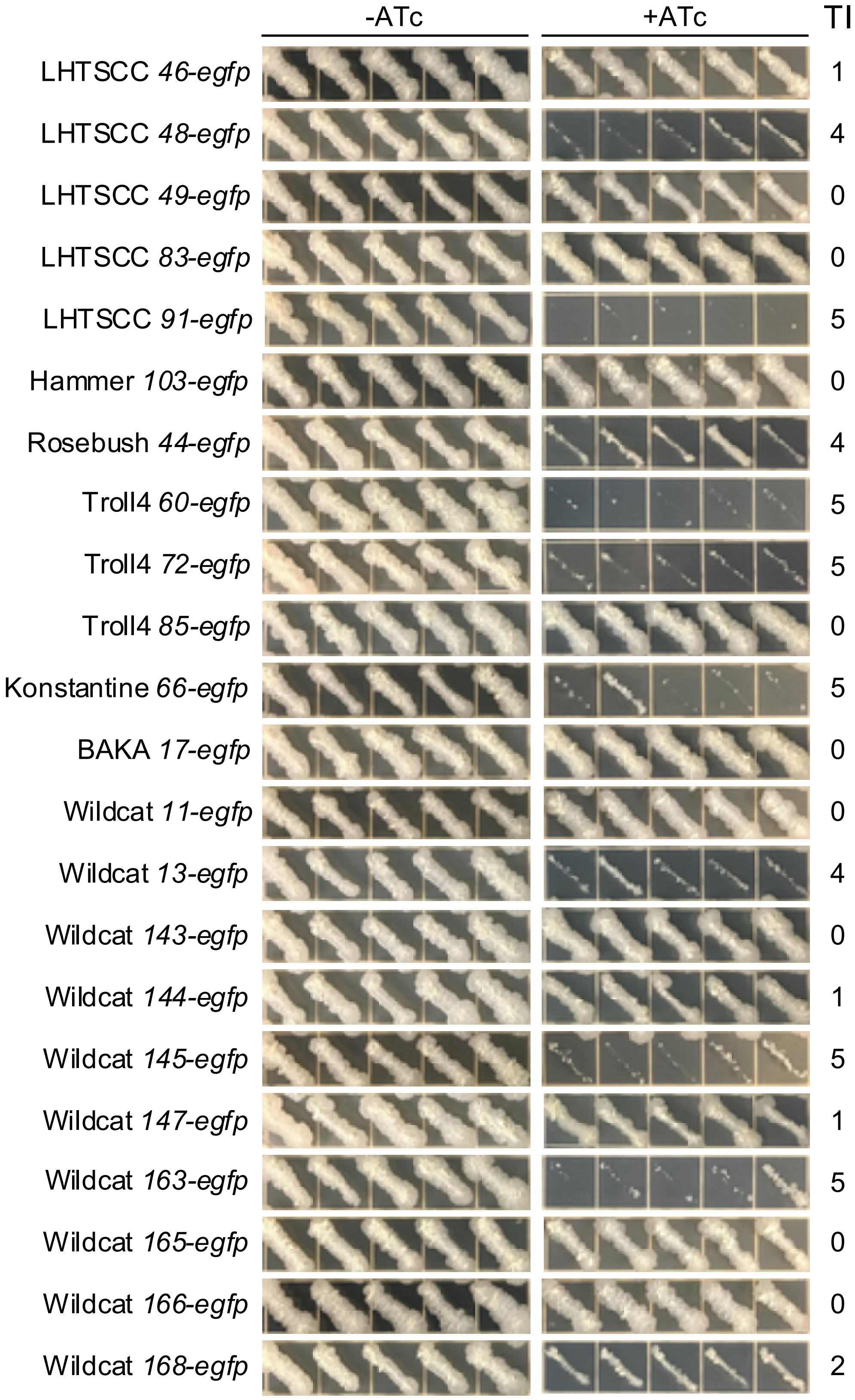

-ATc
+ATc
TI
LHTSCC 46-egfp 1
LHTSCC 48-egfp 4
LHTSCC 49-egfp 0
LHTSCC 83-egfp 0
LHTSCC 91-egfp 5
Hammer 103-egfp 0
Rosebush 44-egfp 4
Troll4 60-egfp 5
Troll4 72-egfp 5
Troll4 85-egfp 0
Konstantine 66-egfp 5
BAKA 17-egfp 0
Wildcat 11-egfp 0
Wildcat 13-egfp 4
Wildcat 143-egfp 0
Wildcat 144-egfp 1
Wildcat 145-egfp 5
Wildcat 147-egfp 1
Wildcat 163-egfp 5
Wildcat 165-egfp 0
Wildcat 166-egfp 0
Wildcat 168-egfp 2

Figure S7

MOTIF 1

```
                                                                              Genes      Coordinates
     **** ********** *   * *******  *  ***** *****  ******* ** ***
5'-GTGAAAGCCCATGGGACACAACGTGTCCGGTTAGGTACTTCAAACGTACCCATGGGCACTAGGCAGCA   162-163  73281. .73343
5'-CCGAAAGCCCATGGGACACAACGTGTCCGGTCAGTCACTTCGAACGTGACCATGGGTACTAGGCAATG   168-169  75641. .75703
5'-CCGAAAACCCATGGGACGCAATGCGTCCGGTAAGGCACTTCAAACGTGCCCATGGGTACGAGGCTCAA   163-164  73751. .73813
5'-ACGAAAACCCATGGGACGCTTAGCGTCCGGTCAGCTACTTCGAACGTAGCCATGGGTACTGAGACACC   164-165  74180. .74242
```

MOTIF 2

```
                                                            Genes     Coordinates
      ****** ********** *************    * ** *****
5'-CGTATGTGTGGGTGGTAGGCCATCGAAGCAACACGGGACACTGGGTAACG  166-167  74828. .74909
5'-TTCATGTGTAGGTGGTAGGCAATCGAAGCAACACAGTGCGCTAGGTAATC  167-168  75213. .75294
```

Figure S8

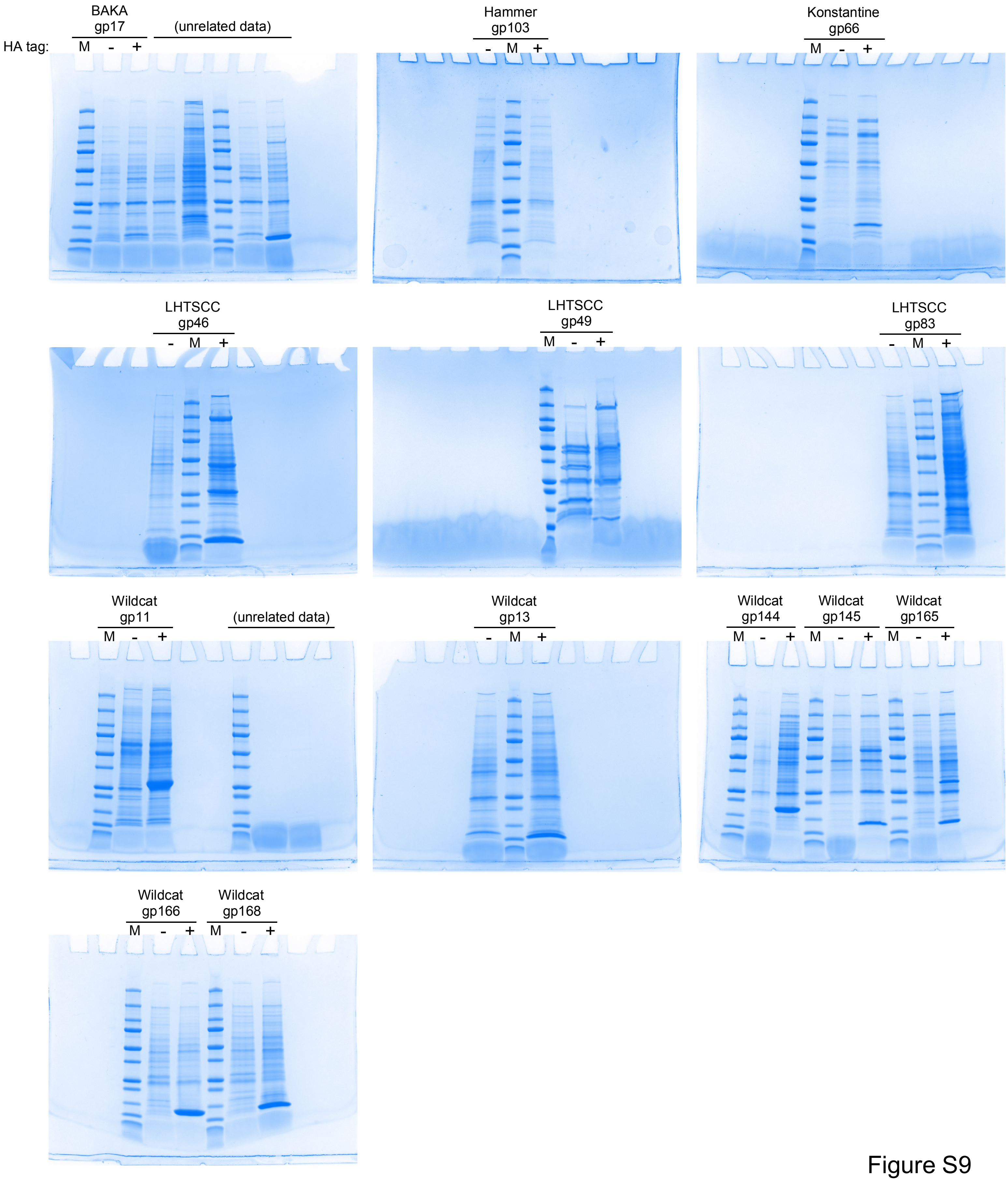
BAKA
gp17
(unrelated data)
HA tag:
M - +
Hammer
gp103
- M +
Konstantine
gp66
M - +
LHTSCC
gp46
- M +
LHTSCC
gp49
M - +
LHTSCC
gp83
- M +
Wildcat
gp11
M - +
(unrelated data)
Wildcat
gp13
- M +
Wildcat
gp144
M - +
Wildcat
gp145
M - +
Wildcat
gp165
M - +
Wildcat
gp166
M - +
Wildcat
gp168
M - +

Figure S9
